# NT5DC2 inhibits ferroptosis by stabilizing ACSL3 in bladder cancer

**DOI:** 10.1038/s41420-026-03091-1

**Published:** 2026-04-14

**Authors:** Shaorui Niu, Pang Yang, Yuyang Yao, Xiaofeng Tang, Jun Yang, Feifei Zhang, Kangming Chen, Chengli Jiang, Yuhao Zhou, Wei Bai, Liping Li, Yuntong Zhou, Xiao-Bin Lv

**Affiliations:** 1 Jiangxi Key Laboratory of Oncology, The Central Lab of The First Hospital of Nanchang, The Third Affiliated Hospital, Jiangxi Medical College, Nanchang University, Nanchang, China; 2 Department of Urology, The First Hospital of Nanchang, The Third Affiliated Hospital, Jiangxi Medical College, Nanchang University, Nanchang, China; 3 Zhongshan School of Medicine, Sun Yat-Sen University, Guangzhou, China

**Keywords:** Bladder cancer, Cancer epigenetics

## Abstract

Ferroptosis, a form of regulated cell death, plays a pivotal role in the development and treatment of cancer because of its impact on tumor cell proliferation, differentiation, and resistance to chemotherapy. NT5DC2, a gene associated with ferroptosis, has been identified as a key facilitator of cellular proliferation and metastasis in several cancers. In this study, we found that NT5DC2 is highly expressed in bladder cancer tissues compared with normal tissues and that its expression is correlated with the poor prognosis of bladder cancer patients. Functionally, we demonstrated that NT5DC2 suppresses ferroptosis in bladder cancer cells and promotes malignant tumor progression. Mechanistically, NT5DC2 interacts with ACSL3 and hampers its ubiquitination, thereby improving the stability of ACSL3, a crucial ferroptosis suppressing protein induced by oleic acid in lymph nodes. In addition, rescue assay results indicated that ACSL3 mediated the roles of NT5DC2 in suppressing ferroptosis of bladder cancer cells. Furthermore, we found that the upregulation of ACSL3 by oleic acid treatment was mediated by NT5DC2 as manifested by the observation that silencing of NT5DC2 abrogates this regulatory effect of oleic acid treatment. Collectively, our findings suggest that NT5DC2/ACSL3 plays a critical role in bladder cancer progression and ferroptosis regulation, suggesting that NT5DC2/ACSL3 is a potential therapeutic target for bladder cancer treatment.

## Introduction

Bladder cancer (BLCA) is the 10th most prevalent cancer, with approximately 573,000 new diagnoses and 212,000 cancer-related fatalities worldwide in 2020 [1]. The prevalence of bladder cancer is higher in men than in women, with the majority of patients being over 60 years of age [2]. Upon initial presentation, 70–75% of patients present with nonmuscle invasive bladder cancer (NMIBC), 20–25% present with muscle invasive bladder cancer (MIBC), and 5% are diagnosed with metastatic illness [3, 4]. Conventional therapies for BLCA mostly include surgery, chemotherapy, radiation, and immunotherapy [5]. Despite significant advancements in BLCA treatment, the overall prognosis for patients remains unfavorable due to drug resistance, metastasis, and other complications [6]. Consequently, additional studies are necessary to investigate the potential genetic underpinnings of bladder tumor development and progression, the results of which may facilitate the identification of diagnostic and prognostic biomarkers to inform the clinical management of bladder tumors.

Ferroptosis was initially recognized as a unique iron-dependent process of nonapoptotic regulated cell death characterized by lipid peroxidation and the accumulation of reactive oxygen species [7, 8]. Prevalent pathways of ferroptosis include dysregulated iron metabolism, lipid peroxidation, and the glutathione system [8]. In addition, ferroptosis suppressor protein a (FSP1)/CoQ10 represents a novel axis of ferroptosis that is capable of reducing CoQ10 to CoQ10 H2, thereby functioning as an antioxidant, diminishing intracellular lipid peroxidation, and inhibiting intracellular ferroptotic cell death [9]. Numerous studies indicate that targeting ferroptosis may serve as a viable treatment strategy across various tumor types. A recent study indicated that CST1 expression safeguards gastric cancer cells from ferroptosis, hence facilitating their development and metastasis [10]. Moreover, METTL17 mediates a protective mechanism for cellular survival and ferroptosis in mitochondria, suggesting that METTL17 is a prospective therapeutic target for colorectal cancer (CRC) [11]. Previous studies have revealed that YTHDC1 suppresses lung cancer tumor development and regulates ferroptosis by altering FSP1 mRNA stability, suggesting the potential for ferroptosis-related lung cancer treatment [12]. Although various cancer-related signaling pathways and genes regulate ferroptosis in cancer cells and certain ferroptosis inducers show potentially clinical value in treating cancers, the exact mechanisms of ferroptosis in specific cancer types are not completely clear.

5′-Nucleotidase domain containing 2 (NT5DC2) is a constituent of the haloacid dehalogenase-type phosphatase NT5DC family and has the ability to facilitate the breakdown of nucleotides within cells [13, 14]. Recent findings indicate that the NT5DC2 protein may play a significant role in carcinogenesis [15]. In soft tissue sarcoma, low NT5DC2 expression predicts a good prognosis and inhibits cell development via the ECM‒receptor interaction pathway [16]. By blocking the EGFR pathway, NT5DC2 knockdown slows triple-negative breast cancer development, glycolysis, and neuropathic pain [17]. NT5DC2 knockdown restrained cell proliferation and induced apoptosis in NSCLC cells, and these effects are almost completely eliminated by downregulating p53 expression, indicating that p53 is required for NT5DC2-regulated cell proliferation and apoptosis in NSCLC cells [18].

Cancer cell proliferation and lipid utilization depend on acyl-CoA synthetase long-chain family member 3 (ACSL3), which activates extracellularly generated long-chain fatty acids [19]. ACSL3 significantly enhances the activation of monounsaturated fatty acids (MUFAs), thereby reducing reactive oxygen species (ROS) production by incorporating MUFAs into membrane phospholipids in place of polyunsaturated fatty acids (PUFAs), ultimately altering membrane properties [20, 21]. Research has demonstrated that oleic acid enhances the ability of melanoma cells to metastasize while simultaneously protecting them from ferroptosis in an ACSL3-dependent manner [22]. In addition, by utilizing ACSL3, mammary adipocytes inhibit oleic acid-induced ferroptosis in breast cancer cells [23]. However, the exact mechanism by which oleic acid treatment upregulates ACSL3 remains unknown.

In this study, we revealed that NT5DC2 interacts with and stabilizes ACSL3 by modulating its ubiquitination and subsequent degradation, hence influencing bladder cancer biological functions and ferroptosis. Inhibiting NT5DC2 can increase ferroptosis in the bladder, while ACSL3 can reinstate this process. In addition, oleic acid, a crucial inhibitor of ferroptosis, has been found to be increased in the lymph nodes of patients with cancer cell metastasis, increasing the expression of the NT5DC2/ACSL3 axis, which promotes the malignant progression of bladder cancer. Our research elucidated the mechanism of NT5DC2-mediated ferroptosis in BLCA revealing a potential strategy for its treatment.

## Results

### Expression and prognostic value of NT5DC2 in bladder cancer

**Fig. 1:**
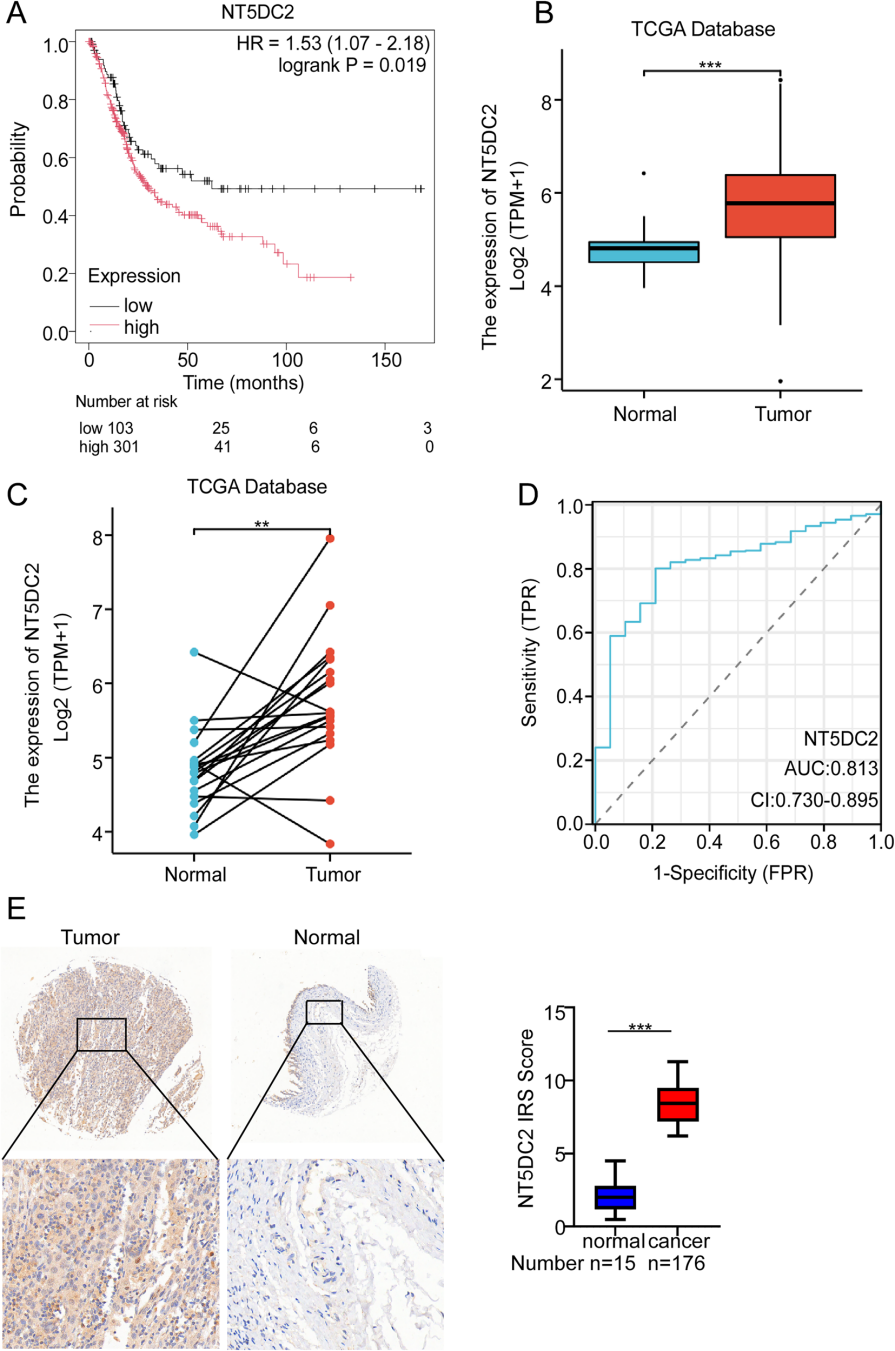
Expression and prognostic value of NT5DC2 in bladder cancer. **A** Overall survival curve from the Kaplan‒Meier analysis. **B** Differential expression of NT5DC2 in BLCA between the tumor group and the normal group. **C** Pairwise differential analysis of NT5DC2 in BLCA. **D** Receiver operating characteristic curve for NT5DC2 expression in normal samples (obtained using GTEx data) and adjoining BLCA tissues and samples. **E** Immunohistochemical analysis of NT5DC2 expression in bladder cancer and adjacent normal tissues and the representative images are shown (scale bars: 50 µm and 100 µm). (*, *P* < 0.05; **, *P* < 0.01; ***, *P* < 0.001).

To identify potential ferroptosis-related genes which may regulate the progression of bladder cancer, we analyzed the differentially expressed genes (DEGs) between bladder cancer and normal tissues from two GEO datasets and intersected the DEGs with the ferroptosis-related gene set. Five genes were identified existing in both DEGs with the ferroptosis-related gene set (Supplementary Fig. 1A). Survival analysis revealed that the expression of NT5DC2 and PPARG, but not the other 3 genes negatively correlated with the overall survival of bladder cancer patients (Fig. 1A and Supplementary Fig. 1B–E). Considering that PPARG was extensively studied in bladder cancer, we chose NT5DC2 for further study. Comparison of the mRNA levels of NT5DC2 in 412 BLCA samples and 19 normal samples through analyzing the TCGA dataset we found that NT5DC2 was obviously upregulated in bladder cancer tissues (Fig. 1B). Additionally, paired comparison between 19 BLCA samples and their matched normal samples showed that NT5DC2 was also upregulated in bladder cancer tissues (Fig. 1C). Next, a receiver operating characteristic (ROC) curve was generated to assess the diagnostic value of NT5DC2 expression. This was accomplished by comparing NT5DC2 expression levels in normal tissue samples (from GTEx) and adjacent BLCA tissues with those in BLCA samples. The results revealed an area under the curve (AUC) value of 0.813 (confidence interval = 0.730–0.895), suggesting that NT5DC2 expression has strong diagnostic potential (Fig. 1D). Immunohistochemical results revealed that the NT5DC2 expression was significantly higher in cancer tissues compared with the normal tissues (Fig. 1E). These results collectively indicated that NT5DC2 related the progression of bladder cancer.

### NT5DC2 increases BLCA cell proliferation, migration and invasion

**Fig. 2:**
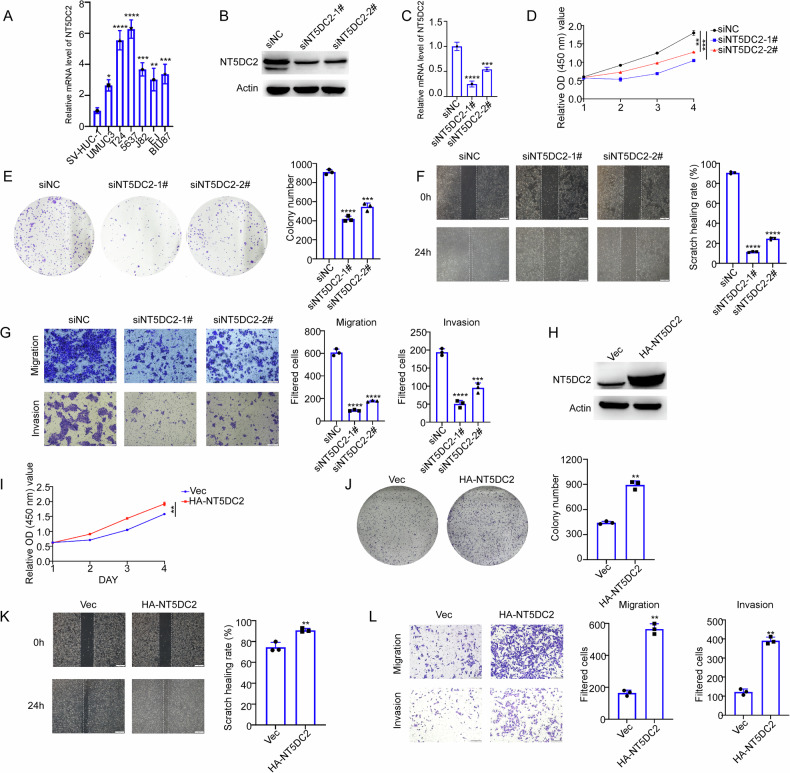
NT5DC2 increases BLCA cell proliferation, migration and invasion. **A** The NT5DC2 mRNA expression in BLCA cell lines(SV-HUC-1, UMUC3, T24, 5637, J82, EJ and BIU87) was quantified by qRT-PCR. 5637 cells transfected with siNC, siNT5DC2-1# and siNT5DC2-2# for 72 h were subjected to western blot (**B**) and qRT-PCR (**C**). 5637 cells transfected with NT5DC2 siRNAs or NC were subjected to cell viability assay at the indicated time points (**D**) or colony formation assay (**E**). 5637 cells transfected with NT5DC2 siRNAs or NC for 48 h were subjected to wound-healing assay (**F**, scale bars: 100 µm) or transwell assay (**G**, scale bars: 50 µm). **H** UMUC3 cells were transfected with empty vector (Vec) or HA tagged NT5DC2 for 24 h and the overexpression of NT5DC2 was validated by western blot (**H**). UMUC3 cells transfected with NT5DC2 expressing plasmid or vec were subjected to cell viability assay at the indicated time points (**I**) or colony formation assay (**J**). UMUC3 cells transfected with NT5DC2 expressing plasmid or vec for 24 h were subjected to wound-healing assay (**K**, scale bars: 100 µm) or transwell assay (**L**, scale bars: 50 µm). (*, *P* < 0.05; **, *P* < 0.01; ***, *P* < 0.001; ****P < 0.0001; ns not significant).

To examine the functional roles of NT5DC2 in BLCA, we first examined the expression of NT5DC2 in BLCA cells. In line with the BLCA tissues, NT5DC2 expression was also higher in BLCA cell lines (UMUC3, T24, 5637, J82, EJ, and BIU87 cells) than in the immortalized ureteral epithelial SV-HUC-1 cells (Fig. 2A). We knocked down NT5DC2 in 5637 cells using siRNAs (siNC, siNT5DC2-1#, and siNT5DC2-2#) and the knockdown efficiency was examined by qRT-PCR and western blotting (Fig. 2B, C). CCK8 assay results revealed a deceleration in the growth rate of 5637 cells following NT5DC2 knockdown (Fig. 2D); colony formation assays produced comparable findings (Fig. 2E). In addition, wound healing and transwell assays indicated that NT5DC2 knockdown reduced the migratory and invasive abilities of 5637 cells (Fig. 2F, G). In contrast, NT5DC2 overexpression in UMUC3 cells increased their proliferation, colony formation, and migratory and invasive abilities (Fig. 2H–L). In summary, these results indicate that NT5DC2 can affect the proliferation, migration, and invasion of BLCA cells.

### NT5DC2 inhibits ferroptosis in BLCA

**Fig. 3:**
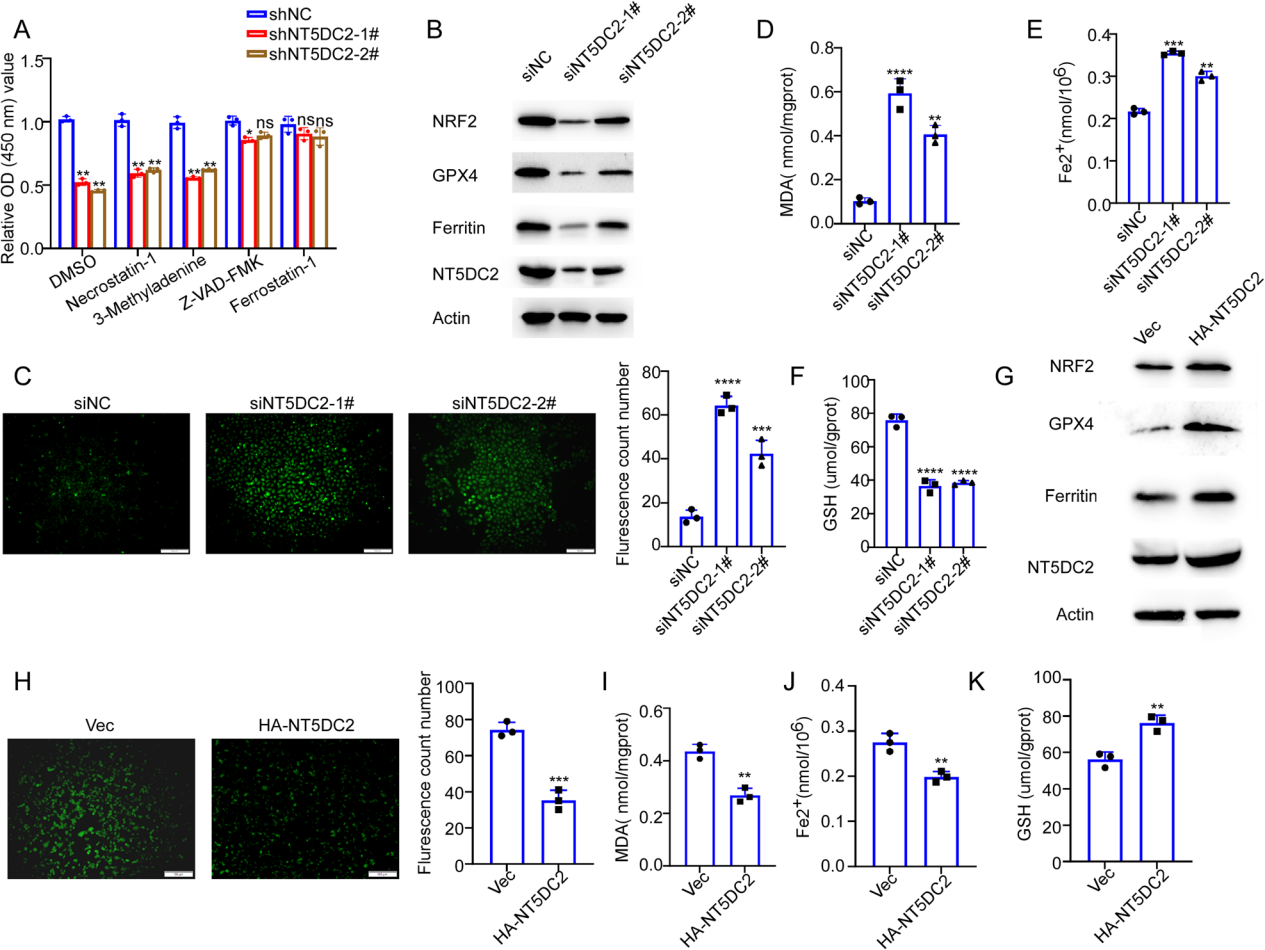
NT5DC2 inhibits ferroptosis in BLCA. **A** CCK-8 assay was used to detect the viability of 5637 cells after transfection with shNC, shNT5DC2-1#, or shNT5DC2-2# and treatment with different cell death inhibitors. Cells were treated with DMSO (control), Necrostatin-1 (10 μM, necroptosis inhibitor), 3-Methyladenine (1 mM, autophagy inhibitor), Z-VAD-FMK (10 μM, apoptosis inhibitor), or Ferrostatin-1 (5 μM, ferroptosis inhibitor) for 48 h before detection. **B** Western blot analysis of ferroptosis-related proteins (NRF2, GPX4, and Ferritin) in 5637 cells after transfecting with or without NT5DC2 siRNAs for 72 h. **C** Intracellular ROS levels were detected by immunofluorescence in 5637 cells after transfecting with or without NT5DC2 siRNAs for 72 h (scale bars: 50 µm). **D**–**F** 5637 cells were transfected with NT5DC2 siRNAs or NC for 72 h, and the indicated ferroptosis-related markers were evaluated. **G** Western blot analysis of ferroptosis-related proteins (NRF2, GPX4, and Ferritin) in UMUC3 cells following transfection with Vec or HA-NT5DC2 plasmid. **H** Intracellular ROS levels were detected by immunofluorescence in UMUC3 cells after transfecting with NT5DC2 plasmid for 24 h (scale bars: 50 µm). **I**–**K** UMUC3 cells were transfected with Vec or HA-NT5DC2 plasmid for 24 h, and the indicated ferroptosis-related markers were evaluated. (*, *P* < 0.05; **, *P* < 0.01; ***, *P* < 0.001; ****P < 0.0001).

We subsequently sought to determine the main manner by which silencing of NT5DC2 suppresses the malignant phenotype of BLCA. We performed a rescue experiment using specific inhibitors of different cell death pathways, including Necrostatin-1 (necroptosis inhibitor), 3-Methyladenine (autophagy inhibitor), Z-VAD-FMK (pan-caspase inhibitor), and Ferrostatin-1 (ferroptosis inhibitor). As shown in Fig. 3A, while NT5DC2 knockdown significantly reduced the cell viability compared to the negative control group, Ferrostatin-1 or Z-VAD-FMK treatment restored the viability of NT5DC2-silenced cells to a larger extent than other inhibitors did. These results indicated that silence of NT5DC2 repressed the malignant phenotype of BLCA mainly through promoting apoptosis and ferroptosis. Considering that ferroptosis plays a very important role in the progression of bladder cancer, we sought to further evaluate the role of NT5DC2 on the ferroptosis of BLCA. We found that knocking down NT5DC2 decreased the expression of the ferroptosis-related proteins NRF2, GPX4, and Ferritin (Fig. 3B). In addition, NT5DC2 knockdown increased the ROS, MDA, and Fe^2+^ levels but decreased the GSH levels (Fig. 3C–F). In contrast, NT5DC2 overexpression had the opposite effect on these ferroptosis-related markers (Fig. 3G–K). Taken together, these results indicate that NT5DC2 suppresses BLCA cell ferroptosis.

### Ferrostatin-1 reverses NT5DC2 knockdown-induced functional alterations in bladder cancer cells

**Fig. 4:**
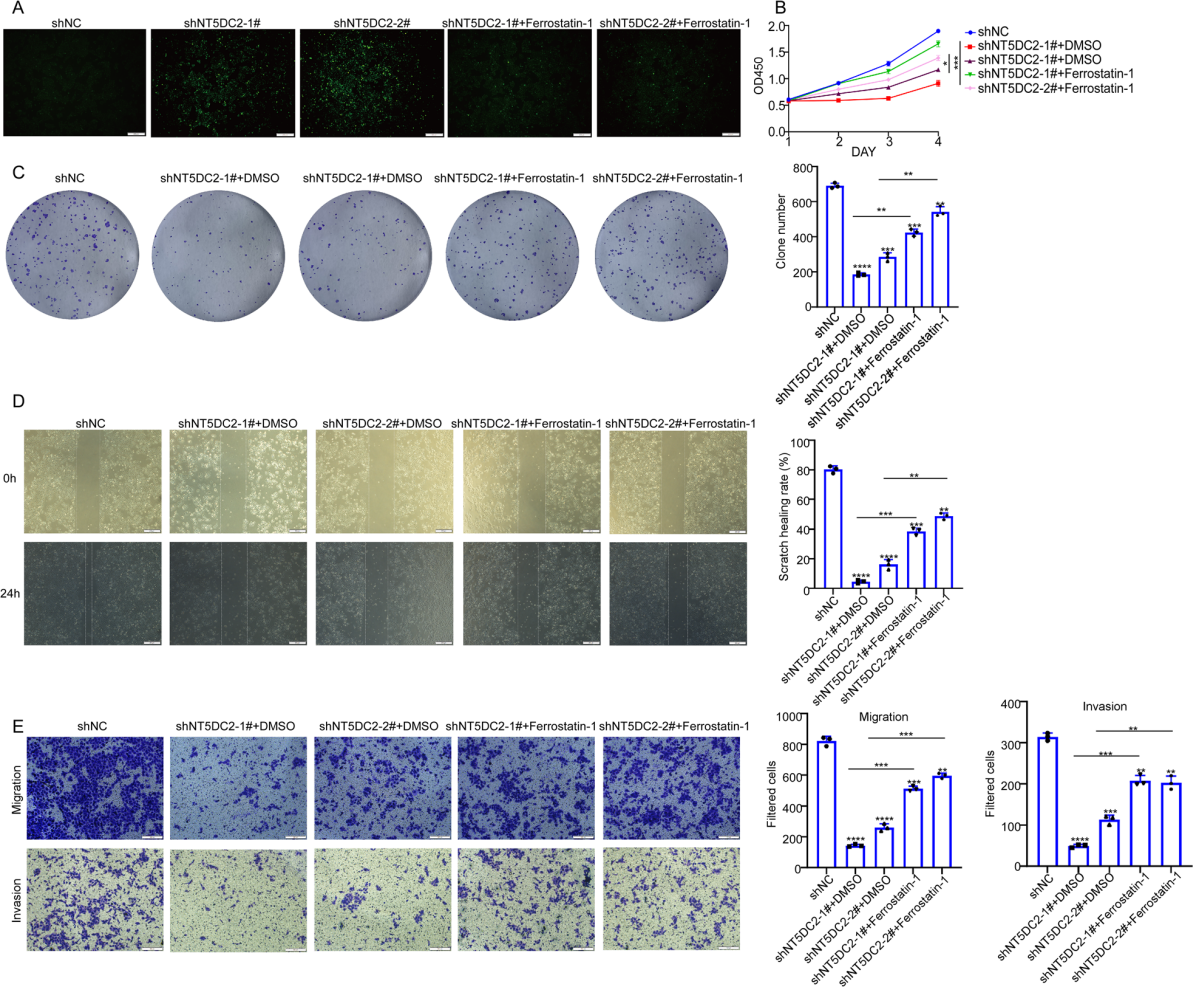
Ferrostatin-1 reverses NT5DC2 knockdown-induced functional alterations in bladder cancer cells. **A** Intracellular ROS levels were detected by immunofluorescence in 5637 cells with stable silencing of NT5DC2 after treatment with DMSO or Ferrostatin-1 (5 μM) for 48 h (Scale bars: 50 µm). **B** 5637 cells with stable silencing of NT5DC2 were treated with DMSO or Ferrostatin-1 (5uM), and cell viability was assessed by CCK-8 assays at 24, 48, 72, and 96 h. **C** 5637 cells with stable silencing of NT5DC2 were treated with DMSO or Ferrostatin-1 (5 μM), and then subjected to colony formation assay. **D** 5637 cells with stable silencing of NT5DC2 were treated with DMSO or Ferrostatin-1 (5 μM), and then subjected to wound-healing migration assay (Scale bars, 100 µm). **E** 5637 cells with stable silencing of NT5DC2 were treated with DMSO or Ferrostatin-1 (5 μM), and then subjected to transwell assay (Scale bars: 50 µm). (*, *P* < 0.05; **, *P* < 0.01; ***, *P* < 0.001; ****P < 0.0001).

We subsequently evaluated whether NT5DC2 regulates the malignant phenotypes of BLCA cells in a ferroptosis-dependent manner. Initially, 5637 cells with NT5DC2 silencing were treated with the ferroptosis inhibitor Ferrostatin-1, with DMSO serving as a control. Immunofluorescence detection of ROS levels showed that NT5DC2 knockdown significantly increased intracellular ROS accumulation, while Ferrostatin-1 treatment effectively reversed this elevation (Fig. 4A), indicating that Ferrostatin-1 works in the BLCA cells. Consistently, CCK-8 assay results indicated an enhancement in the proliferation capacity of NT5DC2-silenced 5637 cells upon Ferrostatin-1 treatment compared to the DMSO control (Fig. 4B). Furthermore, colony formation assay demonstrated that the addition of Ferrostatin-1 partially restored the colony-forming ability of NT5DC2-silenced 5637 cells (Fig. 4C). In terms of cell migration, wound healing assay revealed that Ferrostatin-1 treatment led to a restoration of the migratory capacity of NT5DC2-silenced BLCA cells relative to the DMSO control (Fig. 4D). Similarly, transwell assay confirmed that Ferrostatin-1 administration also rescued the invasive capacity of NT5DC2-silenced cells (Fig. 4E). Taken together, these results indicate that NT5DC2 regulates the malignant traits of BLCA cells partially through inhibiting the ferroptosis.

### NT5DC2 interacts with ACSL3 in BLCA cells

**Fig. 5:**
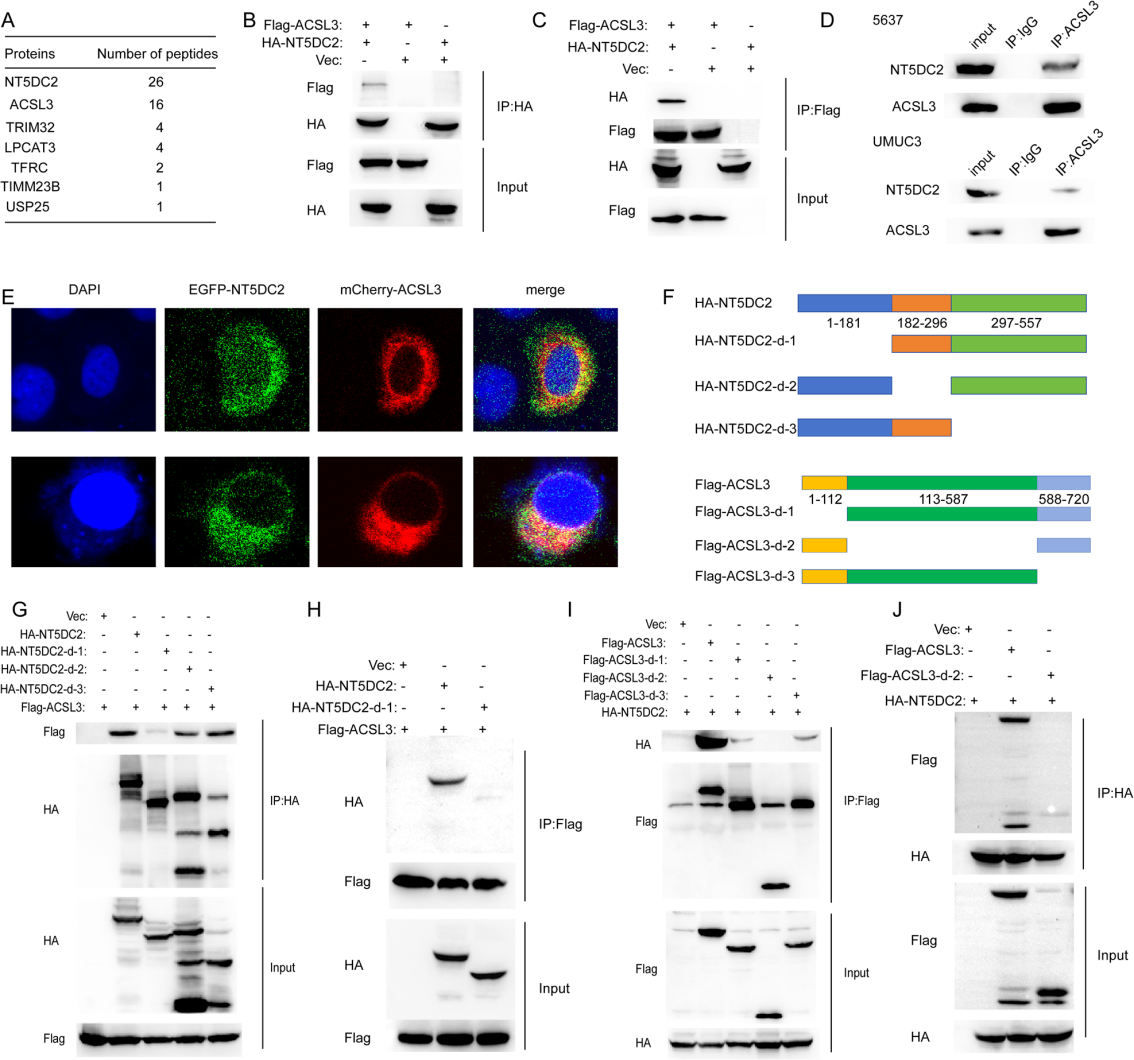
NT5DC2 interacts with ACSL3 in BLCA cells. **A** Lists of the proteins identified in NT5DC2 protein complex by TAP-MS. **B**, **C** 293T cells co-transfected with Flag-ACSL3 and HA-NT5DC2 for 24 h were subjected to co-IP followed by western blotting with indicated antibodies. **D** The indicated cell lysates were immunoprecipitated with an anti-ACSL3 antibody or IgG followed by western blotting with indicated antibodies. **E** 5637 cells cotransfected with GFP-NT5DC2 and ‌mCherry‌-ACSL3 for 24 h were photographed with confocal microscope. Scale bar = 5 µm. **F** Schematic diagram of NT5DC2 and ACSL3 truncation mutants. **G**–**J** 293T cells co-transfected with indicated plasmids for 24 h were subjected to Co-IP followed by western blotting.

To explore the potential mechanisms by which NT5DC2 affects BLCA cell ferroptosis and malignant phenotypes, we performed a tandem affinity purification mass spectrometry (TAP-MS) assay to identify the potential interacting proteins of NT5DC2. We discovered that ACSL3, a crucial ferroptosis-regulating protein, was present with NT5DC2, with high scores (Fig. 5A and Supplementary Fig. 2). Co-IP experiments further validated their interaction at both the exogenous and endogenous levels (Fig. 5B–D). Moreover, the Immunofluorescence (IF) results indicated that NT5DC2 and ACSL3 colocalized in the cytoplasm of 5637 cells (Fig. 5E). To determine the domains that mediate their interaction, we created truncation mutants of both proteins (Fig. 5F). The co-IP results indicated that the NT5DC2 domain comprising amino acids 1-181 interacted with ACSL3 (Fig. 5G, H). In addition, the ACSL3 domain comprising amino acids 113-587 interacted with NT5DC2 (Fig. 5I, J).

### NT5DC2 enhances the stability of ACSL3 and inhibits its ubiquitination

**Fig. 6:**
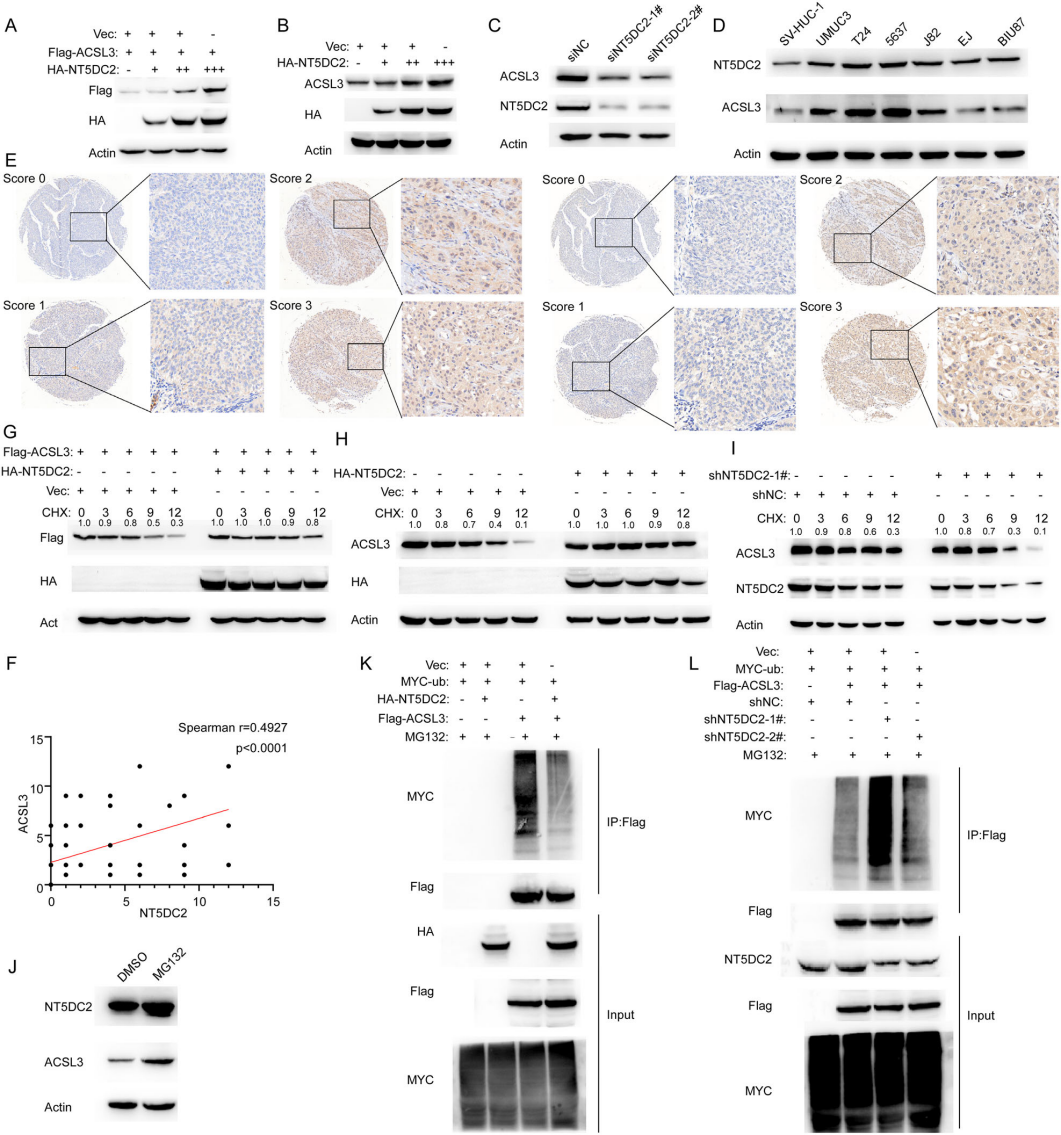
NT5DC2 enhances the stability of ACSL3 and inhibits its ubiquitination. **A** 293T cells transfected with HA-NT5DC2 and increasing quantities of Flag-ACSL3 over 24 h were lysed, and the amounts of the specified proteins were assessed via western blotting. **B** UMUC3 cells were transfected with increasing quantities of HA-tagged NT5DC2 for 24 h, and the levels of the specified proteins were assessed via western blotting. **C** 5637 cells were transfected with NT5DC2 or negative control siRNAs for 72 h, and the levels of the specified proteins were assessed via western blotting. **D** Expression levels of NT5DC2 and ACSL3 in BLCA cell lines (SV-HUC-1, UMUC3, T24, 5637, J82, EJ, and BIU87) were assessed via western blotting. **E** The immunohistochemistry results for NT5DC2 and ACSL3 were scored as follows: SCORE 0, 1, 2, and 3(scale bars: 50 µm and 100 µm). **F** The immunoreactive scores (IRSs) for NT5DC2 and ACSL3. **G** Flag-tagged ACSL3 was cotransfected with or without HA-tagged NT5DC2 into 293T cells for 24 h, after which the cells were treated with CHX for the specified duration prior to harvesting. **H** 5637 cells were transfected with or without HA-tagged NT5DC2 for 24 h, after which the cells were treated with CHX for the specified duration prior to harvesting. **I** 5637 cells with or without stably silencing of NT5DC2 were treated with CHX for the specified duration prior to harvesting. **J** 5637 cells were treated with or without MG132 for 24 h, during which the cells were subjected to CHX treatment for the required duration before harvesting. **K** NT5DC2 overexpression reduced ACSL3 ubiquitination. HA-tagged NT5DC2 was cotransfected into 293T cells with Flag-tagged ACSL3 and Myc-tagged ubiquitin. The ubiquitination of ACSL3 was evaluated using western blotting. **L** Silencing NT5DC2 increased ACSL3 ubiquitination. Flag-tagged ACSL3 and Myc-tagged ubiquitin were cotransfected into 5637 cells with or without NT5DC2 silencing. The ubiquitination of ACSL3 was assessed using western blotting.

We then investigated whether NT5DC2 plays a functional effect on ACSL3. We found that NT5DC2 overexpression markedly increased the exogenous protein levels of ACSL3 in BLCA cells (Fig. 6A). In addition, the regulation of ACSL3 by NT5DC2 at the endogenous protein level was validated in BLCA cells, whereas the mRNA levels of ACSL3 remained unaffected (Fig. 6B, C and Supplementary Fig. 3). Furthermore, the protein levels of NT5DC2 were positively correlated with ACSL3 in BLCA cells (Fig. 6D). More importantly, the immunohistochemical results obtained via a microtissue array containing 176 BLCA tissues revealed a strong correlation between NT5DC2 and ACSL3 protein levels (Fig. 6E, F). Considering that NT5DC2 regulates the protein level of ACSL3, we examined whether NT5DC2 affects the stability of ACSL3. CHX chase assay results indicated that the ectopic expression of NT5DC2 extended the half-life of the ACSL3 protein (Fig. 6G, H), whereas the silencing of NT5DC2 shortened the half-life of the ACSL3 protein (Fig. 6I), indicating that NT5DC2 affects the stability of ACSL3. Previous reports indicate that the expression of ACSL3 is modulated by ubiquitination and is important in renal ischemia‒reperfusion damage and colorectal cancer [24]. To investigate the mechanisms by which NT5DC2 enhances the stability of ACSL3, we assessed whether NT5DC2 affects the ubiquitination of ACSL3. Indeed, MG132, a proteasome inhibitor, increased the protein levels of ACSL3 in BLCA cells (Fig. 6J), indicating that ubiquitination regulates the stability of ACSL3. Additionally, overexpression of NT5DC2 decreased but knockdown of NT5DC2 increased the ubiquitination levels of ACSL3 (Fig. 6K, L). These results collectively indicate that NT5DC2 promotes the expression of ACSL3 in BLCA cells through hampering its ubiquitination-mediated degradation.

### ACSL3 influences the proliferation, invasion, and migration of BLCA cells

**Fig. 7:**
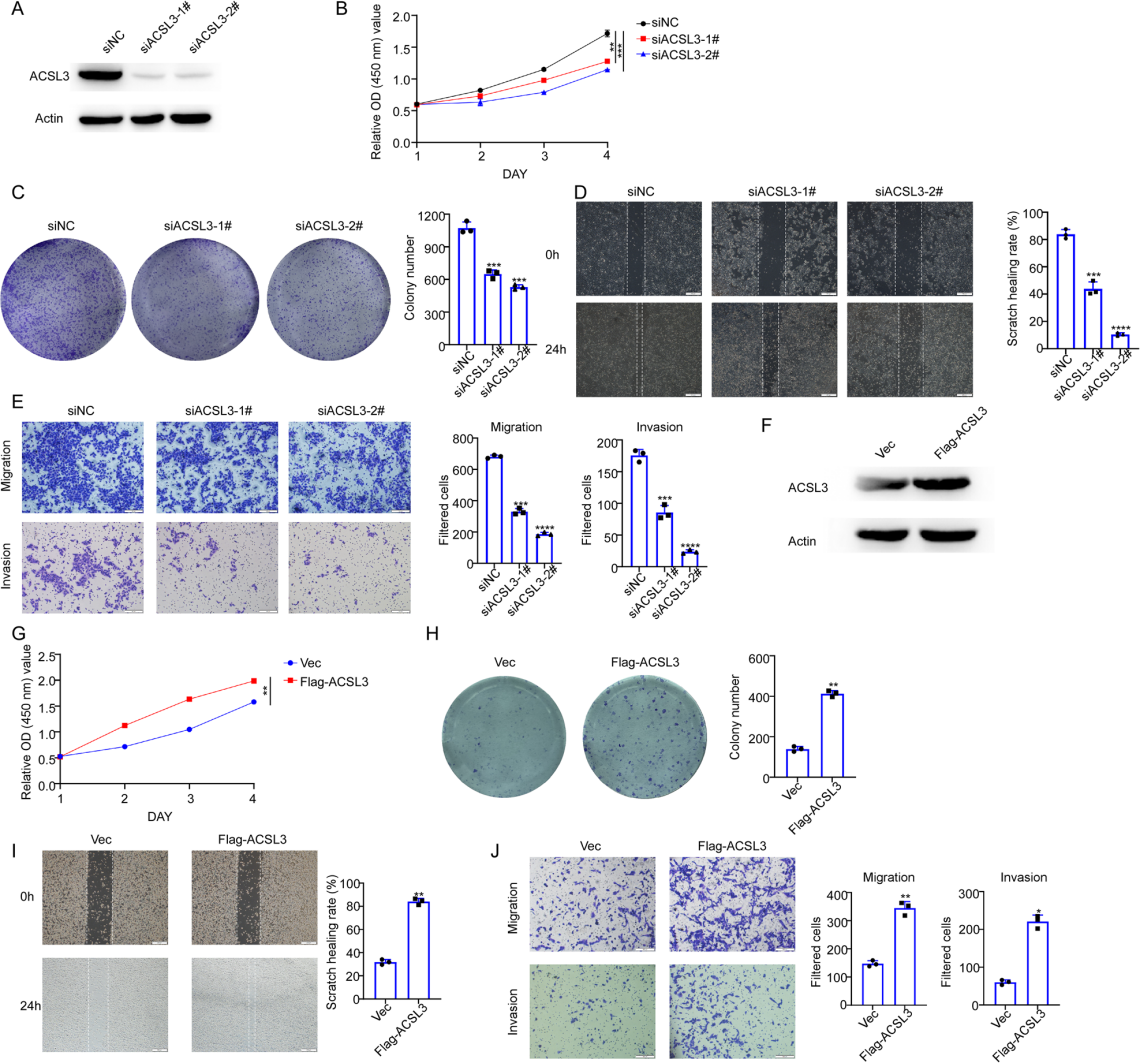
ACSL3 influences the proliferation, invasion, and migration of BLCA cells. **A** 5637 cells were transfected with siNC, siACSL3-1# and siACSL3-2# for 72 h and the knockdown efficiency was determined by western blotting. 5637 cells were transfected with or without ACSL3 siRNAs and the cell viability were examined by CCK-8 (**B**) and colony formatting ability (**C**). 5637 cells were transfected with or without ACSL3 siRNAs for 48 hours were subjected to wound-healing assay and transwell assay (**D**; scale bars: 100 µm) or transwell assay (**E**; scale bars: 50 µm). **F** Validation of ACSL3 overexpression efficiency after transfecting with Vec and Flag-ACSL3 plasmid in UMUC3 cells by western blot. UMUC3 cells were transfected with Vec and Flag-ACSL3 plasmid and the cell viability were examined by CCK-8 (**G**) and colony formatting ability (**H**). **I**, **J** UMUC3 cells were transfected with or without ACSL3 expressing plasmid for 24 h were subjected to wound-healing assay (**I**; scale bars: 100 µm) and transwell assay (**J**; scale bars: 50 µm). (*, *P* < 0.05; **, *P* < 0.01; ***, *P* < 0.001; ****P < 0.0001; ns not significant).

To evaluate whether ACSL3 is a downstream target of NT5DC2, we examined the functional role of ACSL3 in BLCA cells. 5637 cells were transfected with siRNAs targeting ACSL3 (siNC, siACSL3-1#, and siACSL3-2#), and the knockdown efficiency was confirmed through western blot (Fig. 7A). CCK8 assays results revealed a marked reduction in the growth rate of the 5637 cells upon ACSL3 silencing (Fig. 7B). Additionally, silence of ACSL3 obviously reduces the colony formatting ability, migratory and invasive ability of 5637 cells (Fig. 7C–E). On the contrary, overexpression of ACSL3 led to increased cell proliferation, colony formation, migration, and invasion of UMUC3 cells (Fig. 7F–J).

We subsequently examined the roles of ACSL3 on the ferroptosis regulation of BLCA cancer cells. We found that silencing of ACSL3 reduced the protein levels of NRF2, GPX2, and Ferritin, the crucial ferroptosis markers (Supplementary Fig. 4A). Besides, silencing of ACSL3 increased the ROS, MDA and Fe^2+^ levels and reduced the GSH level in 5637 cells (Supplementary Fig. 4B–E). On the contrary, overexpression of ACSL3 in UMUC3 cells obtained the opposite effect (Supplementary Fig. 4F–J). In conclusion, these results indicated that ACSL3 regulates the malignant phenotype and ferroptosis of BLCA cells.

### NT5DC2 regulates the ferroptosis in BLCA cells through ACSL3

**Fig. 8:**
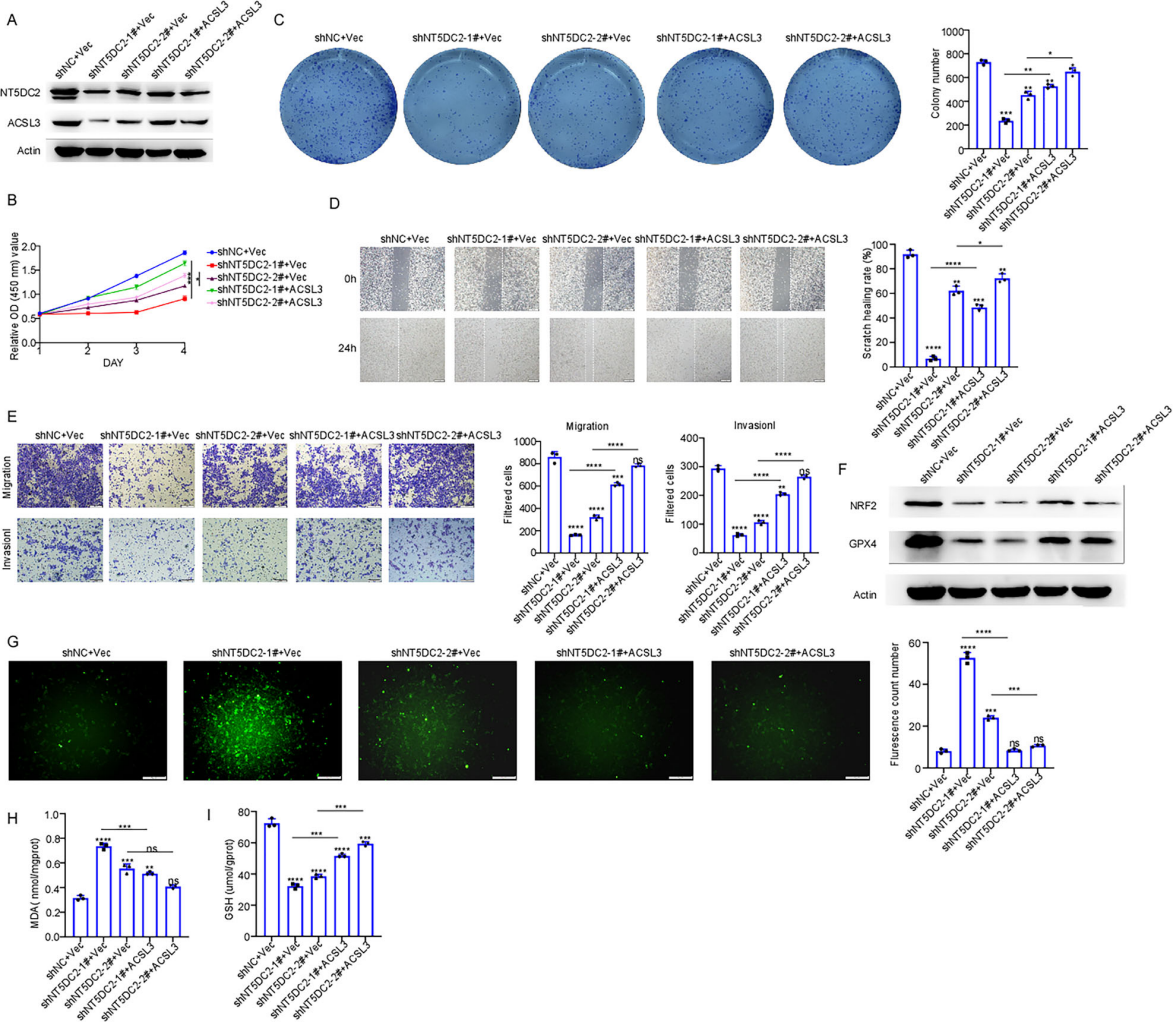
NT5DC2 regulates ferroptosis in BLCA cells through ACSL3. **A** 5637 cells with stable silencing NT5DC2 were transiently transfected with empty vector or ACSL3 for 24 h, and the indicated proteins were examined by western blotting. **B** 5637 cells with stable silencing NT5DC2 were transiently transfected with empty vector or ACSL3, and the cell viability was assessed by CCK-8 assays at 24, 48, 72, and 96 h. 5637 cells with stable silencing NT5DC2 were transiently transfected with empty vector or ACSL3 and then subjected to colony formation assay (**C**) wound-healing migration assay (Scale bars: 100 µm) (**D**) or transwell assay (Scale bars: 50 µm) (**E**). **F** 5637 cells with stable silencing NT5DC2 were transiently transfected with empty vector or ACSL3 for 24 h, and the indicated proteins were examined by western blotting. **G**–**I** 5637 cells with stable silencing NT5DC2 were transiently transfected with empty vector or ACSL3 for 24 h, and the intracellular ROS (Scale bars: 50 µm), MDA and GSH levels were tested. (*, *P* < 0.05; **, *P* < 0.01; ***, *P* < 0.001; ****P < 0.0001; ns not significant).

We subsequently investigated the role of ACSL3 in NT5DC2-mediated ferroptosis suppression in BLCA. A rescue experiment was conducted. CCK-8 and colony formation assays demonstrated that ACSL3 overexpression partially counteracted the inhibitory effects of NT5DC2 knockdown on 5637 cell proliferation (Fig. 8A–C). Additionally, wound healing assays revealed that ACSL3 overexpression restored the migratory capacity of 5637 cells after NT5DC2 knockdown (Fig. 8D). Similarly, ACSL3 overexpression restored both the migratory and invasive abilities of BLCA cells following NT5DC2 silencing (Fig. 8E). Ferroptosis-related analysis revealed that ACSL3 overexpression partially rescued the protein levels of NRF2 and GPX4 after NT5DC2 knockdown (Fig. 8F). Furthermore, ACSL3 overexpression reversed the NT5DC2 knockdown-mediated upregulation of ROS and MDA levels and restored the downregulation of GSH levels (Fig. 8G–J). These findings collectively indicated that ACSL3 mediates the effects of NT5DC2 on BLCA cells.

### NT5DC2 mediated the upregulation of ACSL3 induced by oleic acid

**Fig. 9:**
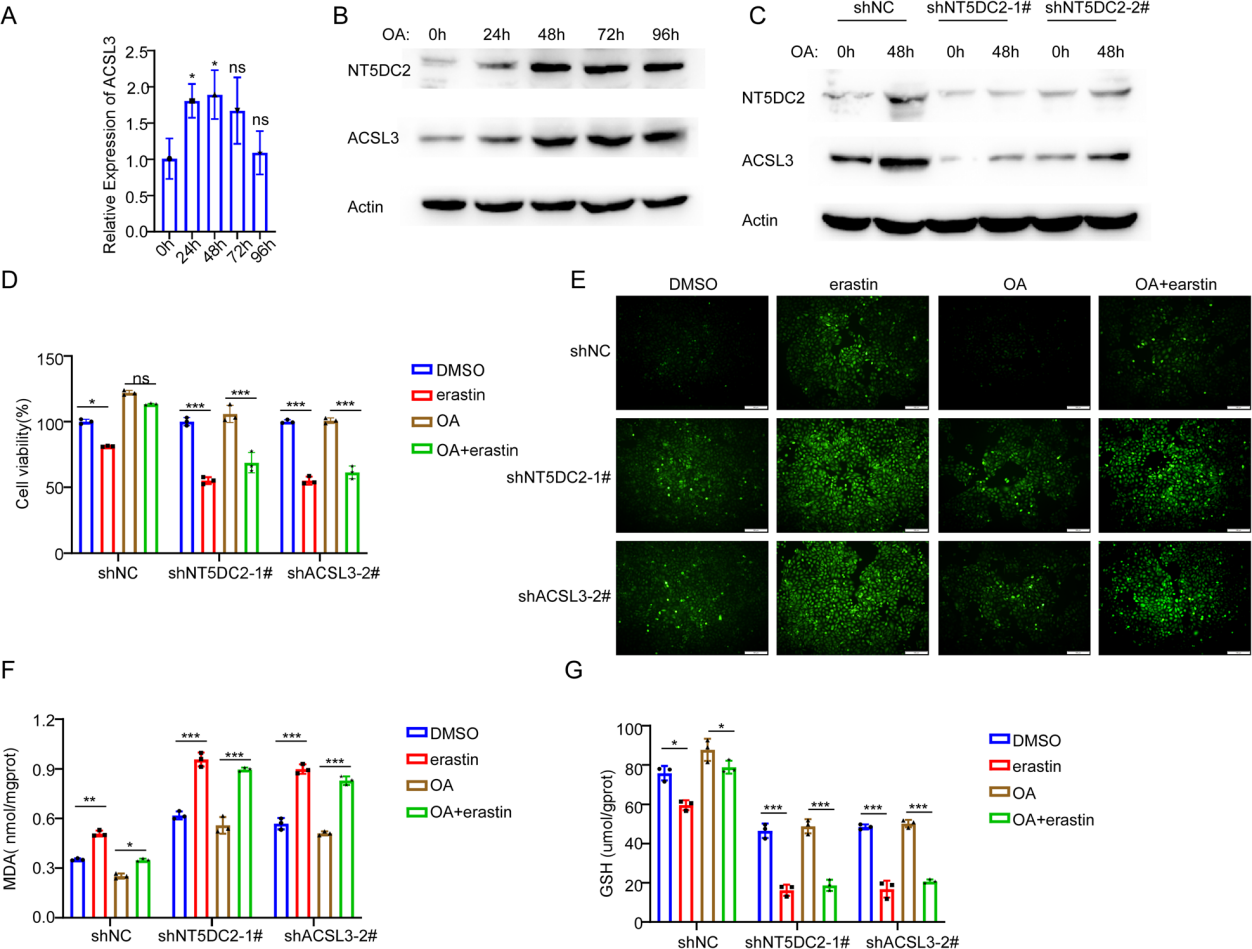
NT5DC2 mediated the upregulation of ACSL3 induced by oleic acid. **A** Quantitative PCR (qPCR) analysis of ACSL3 mRNA levels in 5637 cells treated with oleic acid (200 µM) for 0, 24, 48, 72, or 96 h. **B** Western blotting analysis of NT5DC2 and ACSL3 protein expression in 5637 cells following oleic acid (200 µM) treatment for the indicated durations. **C** Western blotting validation of NT5DC2 and ACSL3 protein levels in shRNA-mediated stable knockdown cell lines (shNT5DC2-1#, shNT5DC2-2#) and the control cell line (shNC) of 5637 cells, after 0 or 48 h of oleic acid treatment. **D** 5637 cells’ stable knockdown cell lines (shNC, shNT5DC2-1#, shACSL3-2#) were treated with the indicated reagents (200 µM oleic acid; 5 µM erastin) for 48 h, and cell viability was assessed using the CCK-8 assay. **E**–**G** 5637 cells’ stable knockdown cell lines (shNC, shNT5DC2-1#, shACSL3-2#) were treated with the indicated reagents (200 µM oleic acid; 5 µM erastin) for 48 h, and the levels of ferroptosis-related markers (ROS, MDA, and GSH) were determined. (*, *P* < 0.05; **, *P* < 0.01; ***, *P* < 0.001; ****P < 0.0001; ns not significant).

Previous studies have demonstrated that oleic acid suppresses ferroptosis through upregulating ACSL3 expression [22, 23]. Based on the above results that NT5DC2 increased the expression of ACSL3, we sought to determine whether oleic acid induces ACSL3 expression through NT5DC2 in BLCA cells. While the mRNA level of ACSL3 was slightly increased, its protein level was increased to a more pronounced extent in BLCA cells after oleic acid treatment, indicating that the upregulation of ACSL3 by oleic acid via NT5DC2 treatment was mainly at the post-translational level. In addition, the NT5DC2 protein was also significantly upregulated at the similar trends as ACSL3 upon oleic acid treatment, suggesting that NT5DC2 might mediate the oleic acid-induced upregulation of ACSL3 in BLCA cells (Fig. 9A, B). Furthermore, silence of NT5DC2 abrogates the upregulation of ACSL3 in BLCA cells upon oleic acid treatment (Fig. 9C). We subsequently investigated whether NT5DC2 mediated the ferroptosis suppression induced by oleic acid. While ferroptosis inducer erastin treatment reduced the viability of 5637 cells, pretreated with oleic acid abrogated this effect mediated by erastin. However, oleic acid pretreatment can’t abrogate erastin treatment-mediated proliferation suppression in either NT5DC2-silenced or ACSL3-silenced 5637 cells (Fig. 9D). The similar effects were also observed in terms of the ferroptosis-related markers including ROS, MDA, and GSH (Fig. 9E–G). These results collectively indicated that NT5DC2 mediated oleic acid-induced ACSL3 upregulation and subsequent ferroptosis suppression in BLCA cells.

### NT5DC2 knockdown decreases BLCA cell xenograft development in vivo

**Fig. 10:**
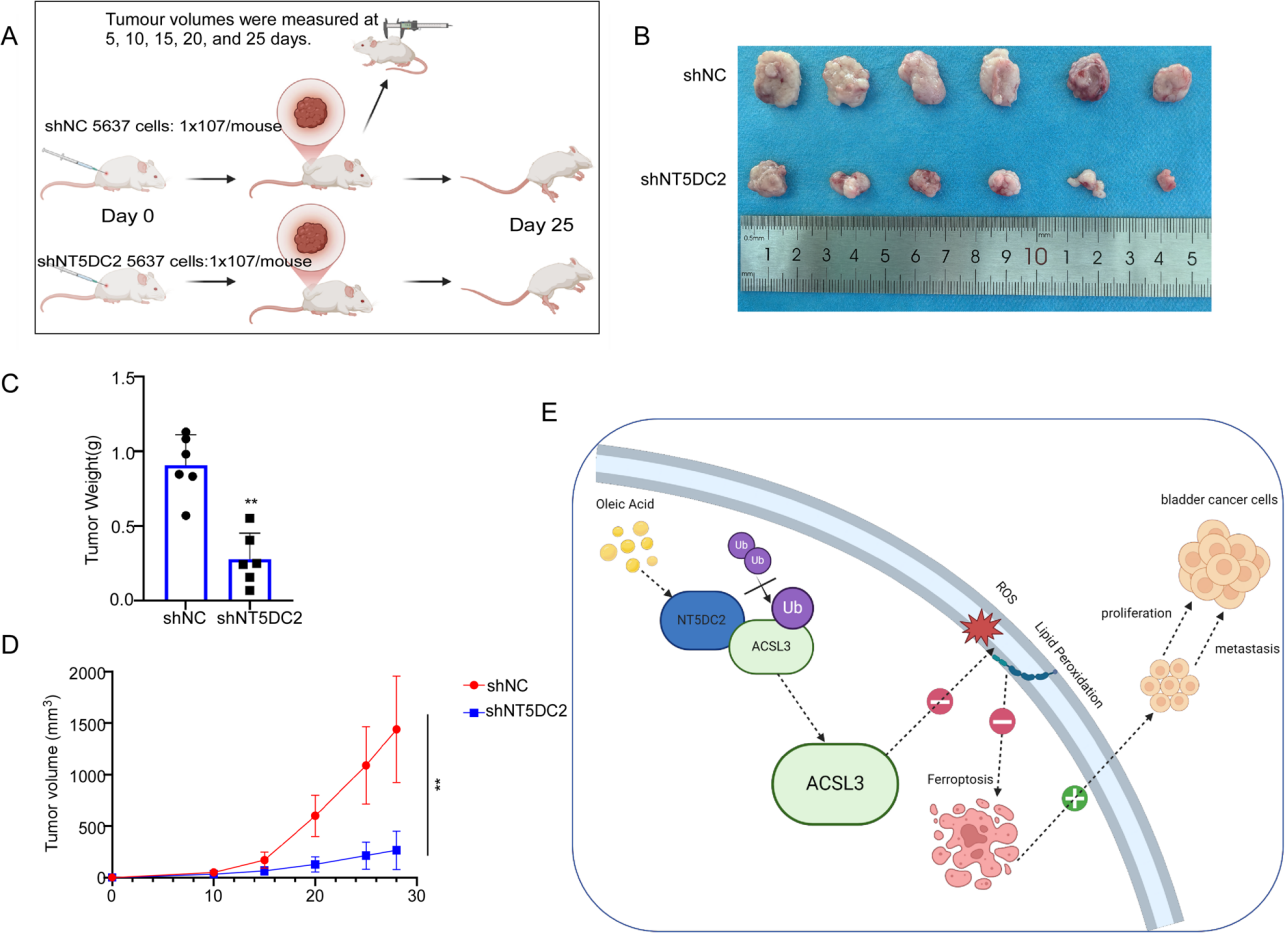
NT5DC2 knockdown decreases BLCA cell xenograft development in vivo. **A** The experimental model diagram was created using Biorender.com. **B** Representative image of a mouse subcutaneous tumor. **C** Measurement of tumor weight after 25 days. **D** Tumor volume was measured every 5 days. **E** The experimental model diagram was created using Biorender.com. **p < 0.01.

To further confirm the functional role of NT5DC2 in BLCA, we performed an in vivo assay. 5637 cells stably silencing NT5DC2 were subcutaneously injected into nude mice (Fig. 10A). The results showed that NT5DC2-silenced xenografts exhibited reduced growth, smaller volume, and lower weight compared with the negative control (Fig. 10B–D). These results indicated that NT5DC2 promotes the proliferation of BLCA in the xenograph model.

## Discussion

Ferroptosis has recently become recognized as a crucial player in cancer diagnosis and treatment because of its impact on tumor cell proliferation, differentiation, and medication resistance in malignant tumors [25, 26]. For example, USP8 inhibition and ferroptosis inducers slow tumor development and increase CD8+ T cell infiltration, which boosts the tumor response to anti-PD-1 immunotherapy [27]. In addition, METTL17-interacting proteins are necessary for mitochondrial gene expression, and their knockdown makes CRC cells susceptible to ferroptosis and limits cell growth [28]. Moreover, the suppression of BAP31 renders gastric cancer cells susceptible to 5-FU and the ferroptosis inducer erastin both in vivo and in vitro [29]. These findings indicate that ferroptosis significantly contributes to carcinogenesis and development, necessitating the aggressive pursuit of ferroptosis-related targets. NT5DC2, a gene associated with ferroptosis, facilitates cellular proliferation and metastasis while diminishing apoptosis in cancers such as sarcoma [16], breast cancer [17], hepatocellular carcinoma [30, 31] and diffuse large B-cell lymphoma [32]. NT5DC2 has been recognized as a possible therapeutic target for the treatment of ferroptosis-related tumors. Nonetheless, the mechanism by which NT5DC2 facilitates the progression of BLCA has not been elucidated. This study provides new mechanistic insights through bioinformatics analysis and experimental confirmation. The oleic acid/NT5DC2/ACSL3 axis was implicated in the malignant biological traits of BLCA (Fig. 9E).

Our study utilized data from both the TCGA and GEO databases and revealed that NT5DC2 expression was higher in BLCA tissues than in normal tissues (Fig. 1). Functional assays involving the knockdown and overexpression of NT5DC2 demonstrated the involvement of NT5DC2 in the malignant progression of BLCA (Fig. 2). Additionally, Kaplan‒Meier plotter analysis revealed a significant association between NT5DC2 expression and the prognosis of patients with BLCA.

Additionally, our research further demonstrated that NT5DC2 inhibits ferroptosis in BLCA cells, thereby promoting tumor progression. Upon NT5DC2 knockdown, the protein levels of NRF2, GPX4, and Ferritin were decreased, whereas the ROS, MDA, and Fe^2+^ levels were increased. In addition, the GSH levels in 5637 cells were decreased. These effects were reversed upon NT5DC2 overexpression (Fig. 3). To further investigate the relationship between NT5DC2 and ferroptosis, treatment with the ferroptosis inhibitor Ferrostatin-1 resulted in a differential restoration of the cell proliferation, migration, and invasion capabilities of bladder cancer cells, which were compromised by ferroptosis induced by NT5DC2 silencing (Fig. 4). On the basis of these observations, we hypothesize that NT5DC2 modulates ferroptosis in BLCA cells. Notably, the core ferroptosis hallmarks observed in our study—including altered Fe^2+^, GSH, ROS, MDA, NRF2, GPX4, and Ferritin—are consistent with the canonical definition of ferroptosis [7, 8]. The rescue effect of Ferrostatin-1 directly indicates that NT5DC2-mediated malignant phenotypes are dependent on ferroptosis [29], which is key experimental evidence for verifying ferroptosis involvement. Our current data have covered the key biochemical and functional characteristics of ferroptosis [33, 34]. However, further evaluating the sensitivity of BLCA cells to ferroptosis inducers following NT5DC2 knockdown (e.g., erastin, RSL3) would further strengthen this conclusion.

To explore the underlying mechanism, mass spectrometry was employed to identify downstream targets of NT5DC2. Our results revealed that NT5DC2 directly interacts with ACSL3 (Fig. 5) and regulates both BLCA cell function and ferroptosis (Fig. 7 and Supplementary Fig. 4). The knockdown and overexpression of NT5DC2 significantly affected the half-life of ACSL3. Moreover, using the proteasome inhibitor MG132, we demonstrated that NT5DC2 regulates ACSL3 protein levels by modulating ACSL3 ubiquitination (Fig. 6). Rescue experiments indicated that the overexpression of ACSL3 could restore BLCA cell function and ferroptosis following NT5DC2 knockdown (Fig. 8).

Given ACSL family, including several members, we analyzed all seven ACSL isoforms via our TAP-MS dataset (Supplementary Table 2). Only ACSL1 and ACSL3 were reliably detected: ACSL3 showed high detection reliability (16 peptides, 27.5% sequence coverage) consistent with our interaction data, while there is only one peptide was identified belong to ACSL1. These results indicate that NT5DC2 specifically targets ACSL3, eliminating confounding effects from other ACSL isoforms on ferroptosis regulation. Of note, ACSL3 overexpression only partially rescued the ferroptotic phenotypes, suggesting the existence of ACSL3-independent pathways. This is consistent with the complex regulatory network of ferroptosis, which involves multiple parallel axes [9]. NT5DC2 has been reported to interact with EGFR and p53 signaling cascades [17, 18], which are closely associated with ferroptosis regulation—for example, p53 can modulate the expression of SLC7A11 (a key component of System Xc⁻) to regulate ferroptosis [12], and EGFR activation can upregulate the stability of GPX4 [32].

Regarding the mechanistic basis of NT5DC2’s effects on NRF2, GPX4, Fe²⁺, and GSH, we propose indirect regulatory mechanisms. As a member of the 5′-nucleotidase family, NT5DC2 is inherently involved in redox homeostasis [13], and its knockdown-induced ROS accumulation can directly inhibit NRF2 transcriptional activity and reduce GPX4 protein stability [26, 33]. In terms of other ferroptosis-related markers, we speculated that the changes of these markers might be an accompanying effect with the process of ferroptosis in BLCA cells.

Previous work has indicated that oleic acid safeguards melanoma cells against ferroptosis in an ACSL3-dependent way and enhances their ability to generate metastatic tumors [22]. Our research revealed that NT5DC2 regulates oleic acid-triggered ACSL3 expression, ultimately inhibiting ferroptosis in bladder cancer cells (Fig. 9). Notably, the suppression of NT5DC2 reduced tumor development in vivo (Fig. 10). Our findings indicated that NT5DC2 promoted the proliferation of BLCA cells.

Nonetheless, our study has several limitations. Initially, while a bioinformatics study revealed a link between NT5DC2 and BLCA prognosis, more confirmation is necessary using materials from BLCA patients. Second, while both in vivo and in vitro experiments have initially validated the role of the oleic acid/NT5DC2/ACSL3 axis in ferroptosis and its influence on the malignant progression of BLCA, further comprehensive investigations are needed to substantiate the findings of this study in future investigations.

## Materials and methods

### Data sources

The GSE37815 and GSE3167 datasets were obtained from the GEO database (https://www.ncbi.nlm.nih.gov/geo/). DEGs were identified using the Limma package (version 3.40.2) of the R program and GEO2R online analysis. False positives in the GEO datasets were rectified using adjusted *p* values. The screening thresholds for DEGs were adjusted *p* < 0.05 and log2 (fold change) (log2|FC|) > 2. A total of 406 genes associated with ferroptosis were obtained from FerrDb (http://www.zhounan.org/ferrdb/current/). Five common DEGs were identified at the intersection of the GSE37815, GSE3167, and FerrDb datasets. GEPIA (http://gepia.cancer-pku.cn/index.html) is an online tool that offers data on 9736 cancer types and 8587 normal samples sourced from TCGA and GTEx [35]. A total of 431 BLCA cases with gene expression data (HTSeq-FPKM) were obtained from TCGA (https://tcga-data.nci.nih.gov/tcga/). Level 3 HTSeq-FPKM data of 431 patients with BLCA were converted into transcripts per million (TPM) values for subsequent analyses. In the TCGA dataset, unidentified clinical characteristics or inaccessible data were considered missing values. The data were categorized into two groups, the NT5DC2 high group and the NT5DC2 low group, on the basis of the median expression level of NT5DC2. A Kaplan–Meier plot database (http://kmplot.com/analysis/, accessed 25 December 2022), which contains 30k+ samples from 21 tumor types, was used to analyze the relationship between NT5DC2 expression and survival in BLCA patients [36]. A correlation was found between NT5DC2 expression and survival in patients with BLCA.

### Cell lines, cell culture, and reagents

SV-HUC-1, UMUC3, T24, 5637, J82, EJ, BIU87 and 293T cells were obtained from the Shanghai Institute of Cell Science, Chinese Academy of Sciences. UMUC3, 5637, J82, EJ and BIU87 cells were cultured in RPMI 1640 medium (Thermo Fisher Scientific, Waltham, MA, USA), SV-HUC-1 cells were cultured in F12K medium (Thermo Fisher Scientific, Waltham, MA, USA), T24 and 293T cells were cultured in DMEM (Thermo Fisher Scientific, Waltham, MA, USA) supplemented with 10% FBS; the cells were maintained at 37 °C in 5% CO_2_. All the cells underwent brief tandem repeat profiling and were cultured for a maximum of 2 months, with all the cell lines confirmed to be mycoplasma free. MG132 (M1902) was purchased from Abmole (Houston, USA). Oleic acid (T2O2668) was purchased from TargetMol Chemicals, Inc. (Shanghai, China). Erastin(571203-78-6) was purchased from MedChemExpress (Monmouth Junction, NJ, USA). Ferrostatin-1(347174-05-4) was purchased from Abmole (Houston, USA). Necrostatin-1(SC4359) and Z-VAD-FMK(C1202) were purchased from Beyotime Biotechnology (Shanghai, China). 3-Methyladenine(A8353) was purchased from APExBIO Technology LLC (Houston, USA).

### RNA extraction and real-time PCR

Total RNA was isolated from cultured cells using TRIzol reagent (Life, USA) in accordance with the manufacturer’s guidelines and subsequently reverse transcribed into cDNA using a PrimeScript™ RT Reagent Kit with gDNA Eraser (Takara, Japan). Quantitative analysis was conducted using RealMaster Mix (SYBR Green Kit, Takara, Shiga, Japan) on a CFX96 Real-Time PCR Detection System (Bio-Rad Laboratories, RRID: SCR_0084b6). RNA levels of target genes were quantified and normalized to those of GAPDH. The primers utilized for quantitative RT–PCR (qRT–PCR) are listed in Supplementary Table 1.

### Cell viability, colony formation ability, wound healing, migration, and invasion assays

Cell viability was assessed at 24, 48, 72, and 96 h. CCK-8 reagent was added to the culture medium, followed by incubation for one hour. The supernatant was collected and transferred to a 96-well plate, where the optical density was assessed at 450 nm. For colony formation assays, 1000 cells per well were seeded in 6-well plates and cultured at 37 °C for 7‒14 days. Fifty cells within a cluster were considered an individual colony. After being fixed with methanol, the cells were stained with 0.1% crystal violet for 30 min, followed by colony imaging and counting. To assess wound healing, a scratch was made using a 200 µl tip in a six-well plate, followed by incubation in FBS-free media. The cells were photographed at 0 and 24 h post-scratch. Matrigel-coated or uncoated transwell chambers (Thermo Fisher Scientific, USA) were used to evaluate cell migration and invasion in vitro. Cells were suspended in 200 µL of serum-free media and placed in upper chambers (5 × 10^4^ for migration, 1 × 10^5^ for invasion). Next, the upper chambers were placed in a 24-well plate, with 600 µL of complete medium in the bottom chamber. Approximately 20 h after treatment, the cells were fixed with methanol and stained with 0.1% crystal violet. After the elimination of unmigrated cells with a cotton swab, ImageJ software was used to quantify the number of migrated cells, and images were taken under a microscope (Olympus, Japan).

### Plasmid or siRNA transfection and the establishment of stable cell lines

NT5DC2 siRNA was acquired from GenePharma (Shanghai). The ACSL3 siRNA sequences were validated [37] and synthesized at GenePharma (Shanghai, China). A scrambled RNA sequence from GenePharma (Shanghai, China) was utilized as negative control for the knockdown experiments. The sequences of the siRNAs used are provided in Supplementary Table 1. siRNA transfection was conducted using Lipofectamine RNAiMAX (Invitrogen, Waltham, MA, USA) in accordance with the manufacturer’s guidelines. Plasmids transfecting into 293T cells were performed using polyethylenimine (PEI; MW = 25,000 Daltons) from Polyscience, Illinois, USA, whereas transfecting into 5637 cells were performed using Lipofectamine 2000 from Invitrogen, Waltham, MA, USA. To produce stable cell lines, 293T cells were cotransfected with pLKO.1-NT5DC2 and packaging/envelope plasmids (psPAX2/pMD2. G) and thereafter incubated at 37 °C for 24 h. The supernatant containing the virus was collected and used to transduce the bladder cancer cells. Following a two-week screening period with puromycin (0.5 µg/ml), the cells were harvested, and the silencing efficiency was assessed.

### Plasmids construction

The S protein-binding peptide, streptavidin-binding peptide, and HA tag were cloned in frame into the pcDNA3.1 vector spanning the BamHI and KpnI endonuclease sites to create the pSSH vector [38]. The full-length NT5DC2 sequence was inserted into the pSSH vector to obtain pSSH-NT5DC2. HA-NT5DC2 and Flag-ACSL3 were purchased from Proteintech (Wuhan, China). The NT5DC2- and ACSL3-truncation plasmids were obtained using a Q5® Site-Directed Mutagenesis Kit (New England Biolabs, USA). NT5DC2 shRNAs were inserted into pLKO.1-puro. The primers used to amplify and clone the recombinant vectors are listed in Supplementary Table 1.

### Western blotting

Cells were lysed in RIPA buffer (50 mM Tris-HCl, 150 mM NaCl, 5 mM EDTA, 0.5% Nonidet P-40) supplemented with a protease inhibitor cocktail (Millipore, 539131-1VL) on ice for 30 min and then centrifuged at 14,000 rpm for 30 min at 4 °C. The supernatants were boiled in 10×SDS loading buffer for 10 min, separated by SDS‒PAGE, and transferred to polyvinylidene fluoride (PVDF) membranes. The membranes were incubated overnight at 4 °C with primary antibodies and for 1 h at room temperature with secondary antibodies.

Following thorough washing, protein bands were visualized using an enhanced chemiluminescence (ECL) kit (Beyotime Ltd., Shanghai, China). The Myc tag (3946 S), HA tag (3724 S) and Flag tag (2368 S) antibodies were purchased from Cell Signaling Technology (Boston, USA). The antibodies against ACSL3 (20710-1-AP) and β-actin (20536-1-AP) used for western blotting were purchased from Proteintech (Wuhan, China). The antibody against NT5DC2 (bs-19491R) used for western blotting was purchased from Bioss (Beijing, China). The antibodies against NRF2 (T55136), GPX4 (T56959) and Ferritin (T55648) used for western blotting were purchased from Abmart (Shanghai, China). The antibody against ACSL3 (30214-1-AP) used for co-IP was obtained from Proteintech (Wuhan, China).

### Coimmunoprecipitation (co-IP)

For the endogenous IP assay, 5637 cells were lysed on ice for 30 min in RIPA lysis buffer and centrifuged at 14000 rpm for 30 min. The supernatant was incubated with 4 μg of anti-ACSL3 antibody at 4 °C for 3 h; IgG was used as the negative control. Following precipitation with protein A/G agarose, the antibody complexes were washed five times with RIPA buffer and boiled for 10 minutes with 10× loading buffer. For the exogenous IP assay, cell lysates were incubated with anti-HA (A2095, Sigma-Aldrich, St. Louis, USA) or anti-Flag (A2220, Sigma-Aldrich, St. Louis, USA) agarose for 4 h or overnight. Next, the agarose was thoroughly washed with RIPA buffer, after which 1× SDS loading buffer was added, and the samples were boiled for 10 min. Proteins were detected via western blotting.

### Ubiquitination assay

HA- or Flag-tagged plasmids were cotransfected with Myc-Ub into 293T cells for 24 h, followed by treatment with MG132 for 6 h before harvesting. After lysing the cells, the supernatant was incubated with anti-HA (A2095, Sigma-Aldrich, St. Louis, USA) or anti-Flag (A2220, Sigma-Aldrich, St. Louis, USA) antibodies for 4 h or overnight. The protein complexes were separated via SDS-PAGE, immunoblotted with an anti-Myc (sc-40AC; Santa Cruz, California, USA) antibody, and, after extensive washing with RIPA buffer, visualized using a chemiluminescence kit.

### Ferroptosis analysis of BLCA cells

To analyze ferroptosis in BLCA cells, the levels of reactive oxygen species (ROS), malondialdehyde (MDA), cellular ferrous iron (Fe^2+^), and reduced glutathione (GSH) were measured. After NT5DC2 was knocked down or overexpressed in BLCA cells, the following tests were performed. ROS levels in BLCA cells were measured using a ROS fluorometric assay kit (E-BC-K138-F, Elabscience), in conjunction with fluorescence microscopy, in accordance with the manufacturer’s instructions. The MDA concentration in BLCA cells was quantified using an MDA colorimetric assay kit (E-BC-K028-M) following the manufacturer’s instructions. Fe^2+^ content was quantified using a cell ferrous iron colorimetric assay kit (E-BC-K881-M, Elabscience). Additionally, the concentration of GSH was measured using a reduced glutathione (GSH) colorimetric assay kit (E-BC-K030-M).

### Immunohistochemistry

A BLCA tissue micro-array (U191BI01) containing 176 BLCA and 15 normal bladder tissues was acquired from Bioaitech Company (Xi’an, China) and subsequently deparaffinized and rehydrated following normal immunohistochemical protocols. Deparaffinized tissue sections were blocked with 3% H2O2 solution, and antigen retrieval was performed with 10 mM citrate buffer at pH 6.0. Following a 30-min blocking period, the sections were incubated with suitably diluted anti-NT5DC2 and anti-ACSL3 antibodies overnight in a humidified chamber at 4 °C. Then, the sections were incubated with sufficiently diluted biotinylated secondary antibodies at room temperature. After one hour of incubation, DAB was used to develop color. Nuclei were visualized via hematoxylin staining. Sections with more than 300 NT5DC2- or ACSL3-positive cells per high-power field (HPF) were defined as strong expression. The expression level of NT5DC2 was semiquantitatively evaluated using the immunoreactive score (IRS), which was calculated as the product of the staining intensity (SI) and the percentage of positive cells (PP). SI was graded as follows: 0, negative; 1, weak; 2, moderate; and 3, strong. PP was categorized as follows: 0, <1%; 1, 1–10%; 2, 11–50%; 3, 51–80%; and 4, >80%. For each tumor, ten different visual fields were examined for IRS assessment. NT5DC2 expression was classified as low when the IRS was ≤4 and high when the IRS was > 4.

### Tumor xenografts

Twelve BALB/c nude mice, aged 4–6 weeks, from the Shanghai Institutes for Biological Sciences in Shanghai, China, were randomly divided into two groups. Nude mice were subcutaneously injected with 5637 cells that were stably transfected with sh-NT5DC2 or sh-NC. Tumor volumes were quantified at specified intervals (5, 10, 15, 20, and 25 days). The mice were euthanized on day 25, and tumors were excised and weighed.

### Statistical analyses

All the experimental data were repeated three times and are presented as means ± SDs. Statistical significance was evaluated using one-way ANOVA for multigroup comparisons and Student’s t-test for two-group comparisons. A difference was deemed significant at *p* < 0.05.

## Supplementary information

supplementary materials

WB-Data

## Data Availability

All the data produced or analyzed in this study are available within the published article and its supplementary information files.
