# Supplementary material for: NT5DC2 inhibits ferroptosis by stabilizing ACSL3 in bladder cancer
Source: Cell Death Discov. 2026 Apr 14;12:235. doi: 10.1038/s41420-026-03091-1 (PMC13184144; doi:10.1038/s41420-026-03091-1)
Supplement: Supplementary file 1 — supplementary materials [file 41420_2026_3091_MOESM1_ESM.pdf]

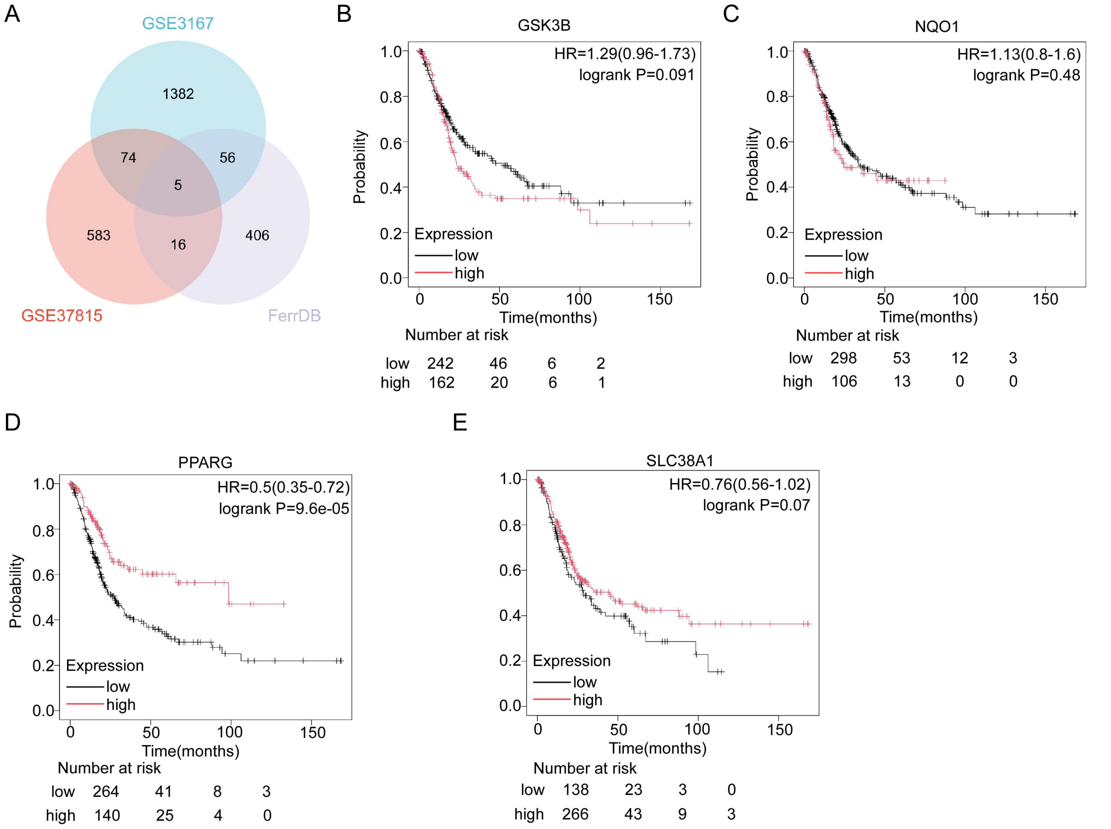

Supplementary 1. Intersection analysis of the GEO and ferroptosis databases revealed NT5DC2 as a potential tumor-promoting gene. (A) Venn diagram illustrating the overlap of genes identified in the GEO database and the ferroptosis database, revealing five intersecting genes. (B) Overall survival curve from the Kaplan–Meier analysis of GSK3B. (C) Overall survival curve from the Kaplan–Meier analysis of NQO1. (D) Overall survival curve from the Kaplan–Meier analysis of PPARG. (E) Overall survival curve from the Kaplan–Meier analysis of SLC38A1.

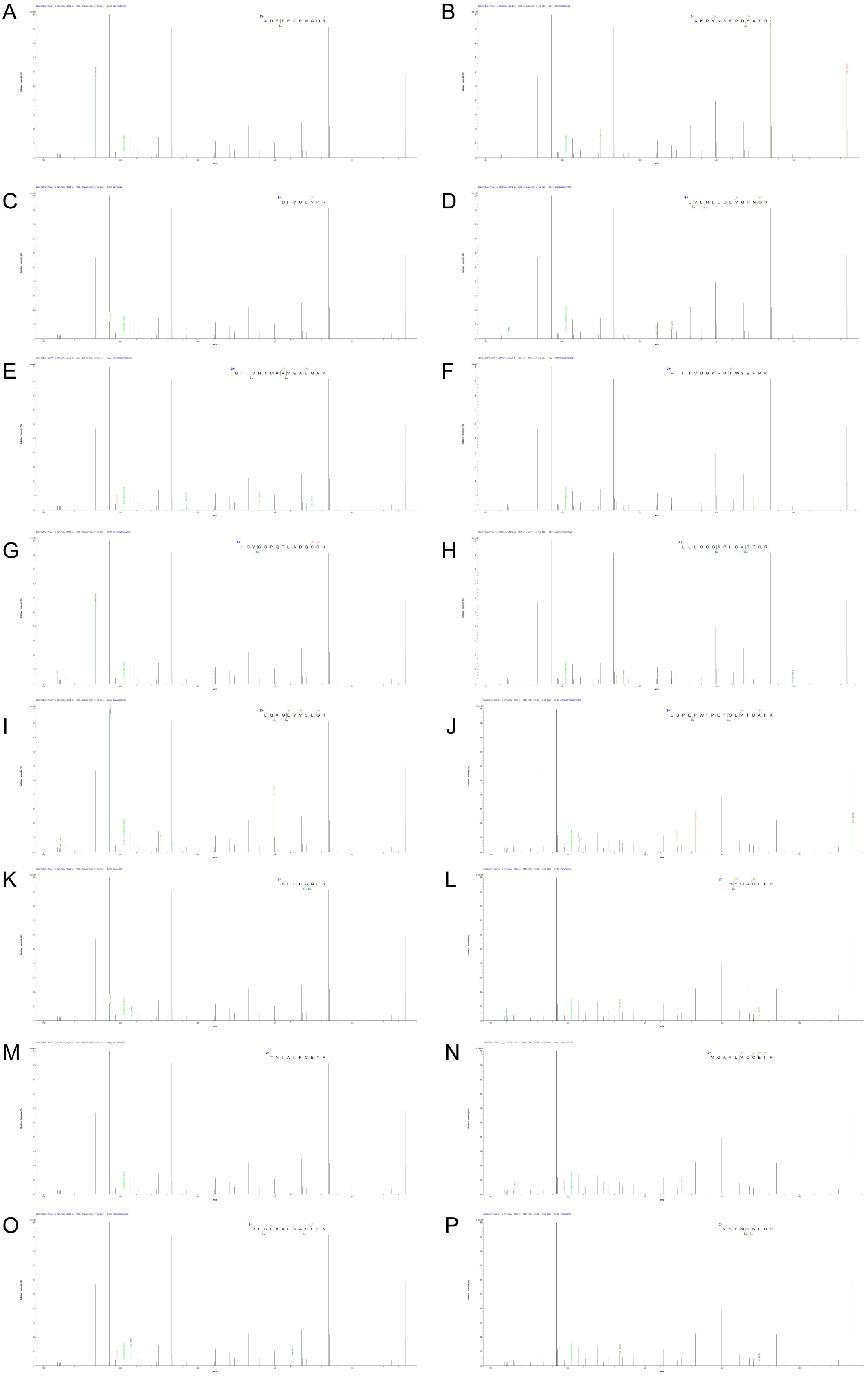

A
ADFFEDENGQR
B
AKPVNSKPDSAYR
C
DIVSLVPR
D
EVLNEEDEVQPNGK
E
GIIVHTMAAVEALGAK
F
HIITVDGKPPTWSEFPK
G
IGYSSPGTLADQSSK
H
LLLCGGAPLSATTQR
I
LQAGEYVSLGK
J
LSPEPWTPETGLVTDAFK
K
SLLGGNIR
L
THYGADIER
M
TNIAIFCETR
N
VGAPLVCCEIK
O
VLSEAAISASLEK
P
VSEMSSFQR

Supplementary 2. Secondary mass spectrum showing the peptides from ACSL3 present in the NT5DC2 protein complex..

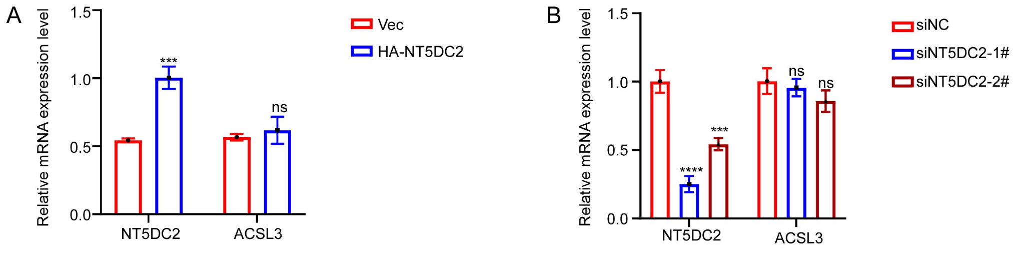

Supplementary 3. NT5DC2 does not affect the mRNA level of ACSL3 in BLCA cells. (A) UMUC3 cells were transfected with an NT5DC2 expression vector for 24 hours (B), or 5637 cells were transfected with NT5DC2 siRNAs for 48 hours, after which the mRNA level of ACSL3 was examined via qRT–PCR (n=3, mean ± SD). Two-tailed unpaired Student's t test was used to determine statistical significance (***, P<0.001; ****P<0.0001; ns, not significant).

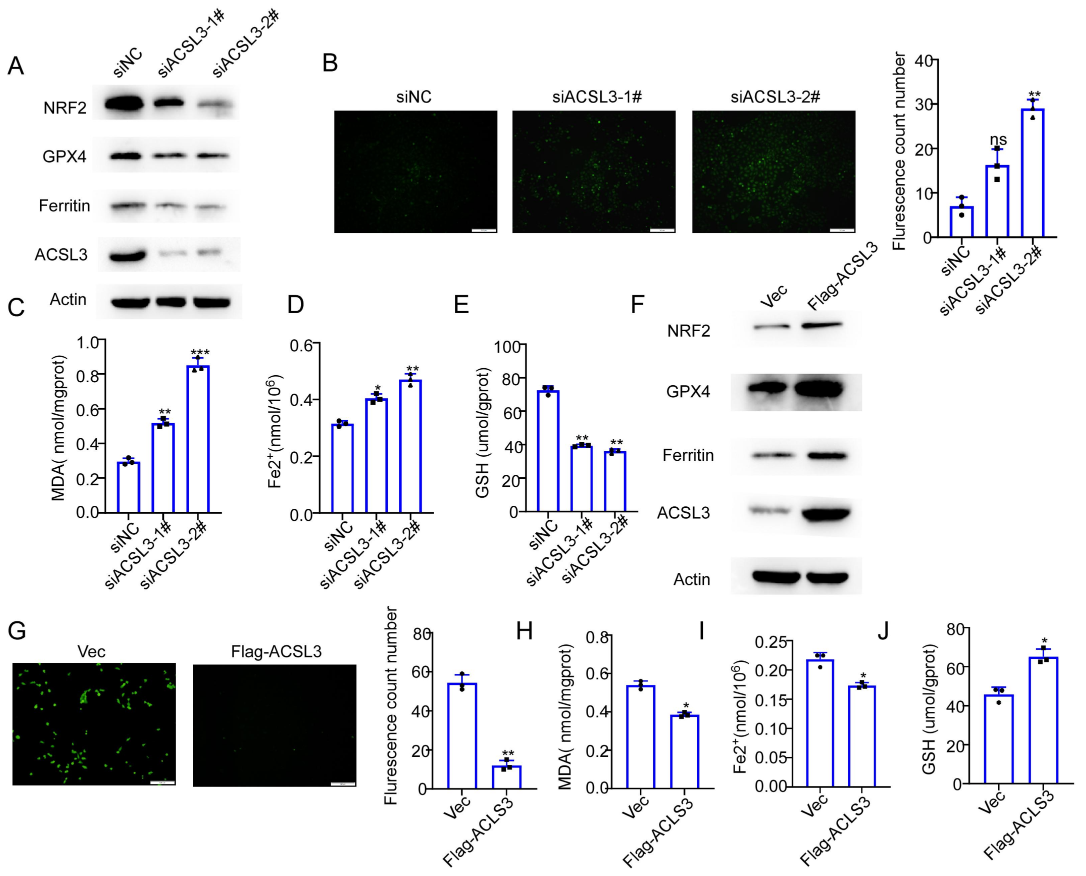

Supplementary 4. ACSL3 inhibits ferroptosis in bladder cancer. (A) Western blot analysis of ferroptosis-related proteins (NRF2, GPX4, and Ferritin) in 5637 cells after transfecting with or without ACSL3 siRNAs for 72 hours. (B) Intracellular ROS levels was detected by immunofluorescence in 5637 cells after transfecting with or without ACSL3 siRNAs for 72 hours (scale bars: 50 μm). (C-E) 5637 cells were transfected with ACSL3 siRNAs or NC for 72 hours, and the indicated ferroptosis-related markers were evaluated. (F) Western blot analysis of ferroptosis-related proteins (NRF2, GPX4, and Ferritin) in UMUC3 cells following transfecting with Vec or Flag-ACSL3 plasmid. (G) Intracellular ROS levels was detected by immunofluorescence in UMUC3 cells after transfecting with Flag-ACSL3 plasmid for 24 hours (scale bars, 50 μm). (H-J) UMUC3 cells transfected withVec or Flag-ACSL3 plasmid for 24 hours, and the indicated ferroptosis-related markers were evaluated. (*P < 0.05; **P < 0.01; ***P < 0.001; ****P < 0.0001; ns, not significant).

Figure 2A qPCR-Data									
	Well	Fluor	Target	Content	Sample	Biological Set Name	Cq	Cq Mean	Cq Std. Dev
	A01	SYBR	GAPDH	Unkn	SV-HUC-1		16.21	16.21	0.000
	A02	SYBR	GAPDH	Unkn	SV-HUC-1		16.28	16.28	0.000
	A03	SYBR	GAPDH	Unkn	SV-HUC-1		16.22	16.22	0.000
	A04	SYBR	GAPDH	Unkn	UMUC3		16.20	16.20	0.000
	A05	SYBR	GAPDH	Unkn	UMUC3		16.43	16.43	0.000
	A06	SYBR	GAPDH	Unkn	UMUC3		16.55	16.55	0.000
	A07	SYBR	GAPDH	Unkn	T24		16.46	16.46	0.000
	A08	SYBR	GAPDH	Unkn	T24		16.39	16.39	0.000
	A09	SYBR	GAPDH	Unkn	T24		16.22	16.22	0.000
	A10	SYBR	GAPDH	Unkn	5637		16.43	16.43	0.000
	A11	SYBR	GAPDH	Unkn	5637		16.32	16.32	0.000
	A12	SYBR	GAPDH	Unkn	5637		16.29	16.29	0.000
	B01	SYBR	GAPDH	Unkn	J82		16.22	16.22	0.000
	B02	SYBR	GAPDH	Unkn	J82		16.22	16.22	0.000
	B03	SYBR	GAPDH	Unkn	J82		16.30	16.30	0.000
	B04	SYBR	GAPDH	Unkn	EJ		16.30	16.30	0.000
	B05	SYBR	GAPDH	Unkn	EJ		16.05	16.05	0.000
	B06	SYBR	GAPDH	Unkn	EJ		16.28	16.28	0.000
	B07	SYBR	GAPDH	Unkn	BIU87		16.30	16.30	0.000
	B08	SYBR	GAPDH	Unkn	BIU87		16.25	16.25	0.000
	B09	SYBR	GAPDH	Unkn	BIU87		16.28	16.28	0.000
	B10	SYBR	NT5DC2	Unkn	SV-HUC-1		23.96	23.96	0.000
	B11	SYBR	NT5DC2	Unkn	SV-HUC-1		24.55	24.55	0.000
	B12	SYBR	NT5DC2	Unkn	SV-HUC-1		24.01	24.01	0.000
	C01	SYBR	NT5DC2	Unkn	UMUC3		22.97	22.97	0.000
	C02	SYBR	NT5DC2	Unkn	UMUC3		22.81	22.81	0.000
	C03	SYBR	NT5DC2	Unkn	UMUC3		22.96	22.96	0.000
	C04	SYBR	NT5DC2	Unkn	T24		21.93	21.93	0.000
	C05	SYBR	NT5DC2	Unkn	T24		21.82	21.82	0.000
	C06	SYBR	NT5DC2	Unkn	T24		21.93	21.93	0.000
	C07	SYBR	NT5DC2	Unkn	5637		21.56	21.56	0.000
	C08	SYBR	NT5DC2	Unkn	5637		21.81	21.81	0.000
	C09	SYBR	NT5DC2	Unkn	5637		21.61	21.61	0.000
	C10	SYBR	NT5DC2	Unkn	J82		22.68	22.68	0.000
	C11	SYBR	NT5DC2	Unkn	J82		22.25	22.25	0.000
	C12	SYBR	NT5DC2	Unkn	J82		23.19	23.19	0.000
	D01	SYBR	NT5DC2	Unkn	EJ		22.23	22.23	0.000
	D02	SYBR	NT5DC2	Unkn	EJ		22.32	22.32	0.000
	D03	SYBR	NT5DC2	Unkn	EJ		20.28	20.28	0.000
	D04	SYBR	NT5DC2	Unkn	BIU87		22.27	22.27	0.000
	D05	SYBR	NT5DC2	Unkn	BIU87		22.57	22.57	0.000
	D06	SYBR	NT5DC2	Unkn	BIU87		22.54	22.54	0.000

Figure 2C qPCR-Data									
	Well	Fluor	Target	Content	Sample	Biological Set Name	Cq	Cq Mean	Cq Std. Dev
	A01	SYBR	GAPDH	Unkn	siNC		17.47	17.47	0.000
	A02	SYBR	GAPDH	Unkn	siNC		17.46	17.46	0.000
	A03	SYBR	GAPDH	Unkn	siNC		17.54	17.54	0.000
	A04	SYBR	GAPDH	Unkn	siNT5DC2-1#		17.33	17.33	0.000
	A05	SYBR	GAPDH	Unkn	siNT5DC2-1#		17.74	17.74	0.000
	A06	SYBR	GAPDH	Unkn	siNT5DC2-1#		17.43	17.43	0.000
	A07	SYBR	GAPDH	Unkn	siNT5DC2-2#		16.50	16.50	0.000
	A08	SYBR	GAPDH	Unkn	siNT5DC2-2#		17.05	17.05	0.000
	A09	SYBR	GAPDH	Unkn	siNT5DC2-2#		16.88	16.88	0.000
	A10	SYBR	NT5DC2	Unkn	siNC		24.34	24.34	0.000
	A11	SYBR	NT5DC2	Unkn	siNC		24.01	24.01	0.000
	A12	SYBR	NT5DC2	Unkn	siNC		24.16	24.16	0.000
	B01	SYBR	NT5DC2	Unkn	siNT5DC2-1#		26.06	26.06	0.000
	B02	SYBR	NT5DC2	Unkn	siNT5DC2-1#		26.37	26.37	0.000
	B03	SYBR	NT5DC2	Unkn	siNT5DC2-1#		26.19	26.19	0.000
	B04	SYBR	NT5DC2	Unkn	siNT5DC2-2#		24.19	24.19	0.000
	B05	SYBR	NT5DC2	Unkn	siNT5DC2-2#		24.63	24.63	0.000
	B06	SYBR	NT5DC2	Unkn	siNT5DC2-2#		24.32	24.32	0.000

Supplement 3A qPCR-Data

	Well	Fluor	Target	Content	Sample	Biological Set Name	Cq	Cq Mean	Cq Std. Dev
	A01	SYBR	GAPDH	Unkn	Vec-NT5DC2		17.47	17.47	0.000
	A02	SYBR	GAPDH	Unkn	Vec-NT5DC2		17.46	17.46	0.000
	A03	SYBR	GAPDH	Unkn	Vec-NT5DC2		17.54	17.54	0.000
	A04	SYBR	GAPDH	Unkn	HA-NT5DC2		16.50	16.50	0.000
	A05	SYBR	GAPDH	Unkn	HA-NT5DC2		17.05	17.05	0.000
	A06	SYBR	GAPDH	Unkn	HA-NT5DC2		16.88	16.88	0.000
	A07	SYBR	GAPDH	Unkn	Vec-NT5DC2		17.50	17.50	0.000
	A08	SYBR	GAPDH	Unkn	Vec-NT5DC2		17.24	17.24	0.000
	A09	SYBR	GAPDH	Unkn	Vec-NT5DC2		17.02	17.02	0.000
	A10	SYBR	GAPDH	Unkn	HA-NT5DC2		17.14	17.14	0.000
	A11	SYBR	GAPDH	Unkn	HA-NT5DC2		17.23	17.23	0.000
	A12	SYBR	GAPDH	Unkn	HA-NT5DC2		17.24	17.24	0.000
	B01	SYBR	NT5DC2	Unkn	Vec-NT5DC2		22.74	22.74	0.000
	B02	SYBR	NT5DC2	Unkn	Vec-NT5DC2		23.01	23.01	0.000
	B03	SYBR	NT5DC2	Unkn	Vec-NT5DC2		23.06	23.06	0.000
	B04	SYBR	NT5DC2	Unkn	HA-NT5DC2		21.19	21.19	0.000
	B05	SYBR	NT5DC2	Unkn	HA-NT5DC2		21.63	21.63	0.000
	B06	SYBR	NT5DC2	Unkn	HA-NT5DC2		21.32	21.32	0.000
	B07	SYBR	ACSL3	Unkn	Vec-NT5DC2		23.17	23.17	0.000
	B08	SYBR	ACSL3	Unkn	Vec-NT5DC2		23.13	23.13	0.000
	B09	SYBR	ACSL3	Unkn	Vec-NT5DC2		23.02	23.02	0.000
	B10	SYBR	ACSL3	Unkn	HA-NT5DC2		23.19	23.19	0.000
	B11	SYBR	ACSL3	Unkn	HA-NT5DC2		23.13	23.13	0.000
	B12	SYBR	ACSL3	Unkn	HA-NT5DC2		22.82	22.82	0.000

Supplement 3B qPCR-Data									
	Well	Fluor	Target	Content	Sample	Biological Set Name	Cq	Cq Mean	Cq Std. Dev
	A01	SYBR	GAPDH	Unkn	siNC		19.31	19.31	0.000
	A02	SYBR	GAPDH	Unkn	siNC		19.07	19.07	0.000
	A03	SYBR	GAPDH	Unkn	siNC		19.20	19.20	0.000
	A04	SYBR	GAPDH	Unkn	siNT5DC2-1#		19.65	19.65	0.000
	A05	SYBR	GAPDH	Unkn	siNT5DC2-1#		19.26	19.26	0.000
	A06	SYBR	GAPDH	Unkn	siNT5DC2-1#		19.12	19.12	0.000
	A07	SYBR	GAPDH	Unkn	siNT5DC2-2#		19.22	19.22	0.000
	A08	SYBR	GAPDH	Unkn	siNT5DC2-2#		19.11	19.11	0.000
	A09	SYBR	GAPDH	Unkn	siNT5DC2-2#		19.22	19.22	0.000
	A10	SYBR	GAPDH	Unkn	siNC		19.11	19.11	0.000
	A11	SYBR	GAPDH	Unkn	siNC		19.17	19.17	0.000
	A12	SYBR	GAPDH	Unkn	siNC		19.32	19.32	0.000
	B01	SYBR	GAPDH	Unkn	siNT5DC2-1#		19.01	19.01	0.000
	B02	SYBR	GAPDH	Unkn	siNT5DC2-1#		19.23	19.23	0.000
	B03	SYBR	GAPDH	Unkn	siNT5DC2-1#		19.28	19.28	0.000
	B04	SYBR	GAPDH	Unkn	siNT5DC2-2#		19.25	19.25	0.000
	B05	SYBR	GAPDH	Unkn	siNT5DC2-2#		19.32	19.32	0.000
	B06	SYBR	GAPDH	Unkn	siNT5DC2-2#		19.11	19.11	0.000
	B07	SYBR	NT5DC2	Unkn	siNC		23.17	23.17	0.000
	B08	SYBR	NT5DC2	Unkn	siNC		23.16	23.16	0.00
	B09	SYBR	NT5DC2	Unkn	siNC		23.11	23.11	0.00
	B10	SYBR	NT5DC2	Unkn	siNT5DC2-1#		25.99	25.99	0.00
	B11	SYBR	NT5DC2	Unkn	siNT5DC2-1#		25.84	25.84	0.00
	B12	SYBR	NT5DC2	Unkn	siNT5DC2-1#		26.21	26.21	0.00
	C01	SYBR	NT5DC2	Unkn	siNT5DC2-2#		24.11	24.11	0.00
	C02	SYBR	NT5DC2	Unkn	siNT5DC2-2#		24.14	24.14	0.00
	C03	SYBR	NT5DC2	Unkn	siNT5DC2-2#		24.10	24.10	0.00
	C04	SYBR	ACSL3	Unkn	siNC		23.17	23.17	0.00
	C05	SYBR	ACSL3	Unkn	siNC		23.16	23.16	0.00
	C06	SYBR	ACSL3	Unkn	siNC		23.11	23.11	0.00
	C07	SYBR	ACSL3	Unkn	siNT5DC2-1#		23.37	23.37	0.00
	C08	SYBR	ACSL3	Unkn	siNT5DC2-1#		23.35	23.35	0.00
	C09	SYBR	ACSL3	Unkn	siNT5DC2-1#		23.32	23.32	0.00
	C10	SYBR	ACSL3	Unkn	siNT5DC2-2#		23.77	23.77	0.00
	C11	SYBR	ACSL3	Unkn	siNT5DC2-2#		23.58	23.58	0.00
	C12	SYBR	ACSL3	Unkn	siNT5DC2-2#		23.48	23.48	0.00

Supplementary Table 1. Information for primers, plasmids and siRNA sequences.

Gene	Forward primer (5'-3')	Reverse primer (5'-3')
**qRT-PCR**		
NT5DC2	CTTCTCGCTACCGGAGATGG	CCGTCACGTCCTTGTAGAGAT
ACSL3	GTTTGGCTTCAGTATTATACCCT	TTCGAGTTCAGTTAGTTCCT
GAPDH	GGAGCGAGATCCCTCCAAAAT	GGCTGTTGTCATACTTCTCATGG
**pSSH vector construction**		
Streptavidin binding peptide sequen	ATGGACGAGAAGACCACCGGCTGGAGAGGCGGCCACGTGGTGGAGGGCCTGGCCGGCGAGCTGGAGCAGCTGAGAGCCAGACTGGAGCACCACCCTCAGGGCCAGAGAGAACCT	
S-protein binding peptide sequence	AAAGAAACCGCTGCTGCTAAATTCGAACGCCAGCACATGGACAGC	
HA-tags	TACCCATACGATGTTCCAGATTACGCT	
**Plasmids construction**		
pcDNA3.1 -HA-S-Streptavidin	AAGGATCCCAGAGTAATAAAACTTTTAACTTGGAG	TTCTCGAGTTAGTATCGGCGACGTTTGT
pLKO.1 -Sh-NT5DC2 -1	CCGGGGAGTTTGACCAAGCACATTTCTCGAGAAATGTGCTTGGTCAAACTCCTTTTT	AATTAAAAAGGAGTTTGACCAAGCACATTTCTCGAGAAATGTGCTTGGTCAAACTCC
pLKO.1 -Sh-NT5DC2 -2	CCGGGCAGGGAAACCTGTTTGACTTCTCGAGAAGTCAAACAGGTTTCCCTGCTTTTT	AATTAAAAAGCAGGGAAACCTGTTTGACTTCTCGAGAAGTCAAACAGGTTTCCCTGC
pLKO.1 -Sh-ACSL3 -1	CCGGGGCCCATGTTCTAGAATTATTCTCGAGAATAATTCTAGAACATGGGCCTTTTT	AATTAAAAAGGCCCATGTTCTAGAATTATTCTCGAGAATAATTCTAGAACATGGGCC
pLKO.1 -Sh-ACSL3 -2	CCGGGGAAGGTGGATACTTTAATTTCTCGAGAAATTAAAGTATCCACCTTCCTTTTT	AATTAAAAAGGAAGGTGGATACTTTAATTTCTCGAGAAATTAAAGTATCCACCTTCC
PSSH-NT5DC2	GTTCCAGATTACGCTGGATCCGCGGGTGCGGGGCTGCG	AACGGGCCCTCTAGACTCGAGTCAGCGGATGTGGGCCATGTCA
**siRNA targeting**		
si-NT5DC2 -1#	GGAGUUUGACCAAGCACAUTT	AUGUGCUUGGUCAAACUCCTT
si-NT5DC2 -2#	GCAGGGAAACCUGUUUGACTT	GUCAAACAGGUUUCCCUGCTT
si-ACSL3 -1#	GGCCCAUGUUCUAGAAUUATT	UAAUUCUAGAACAUGGGCCTT
si-ACSL3 -2#	GGAAGGUGGAUACUUUAAUTT	AUUAAAGUAUCCACCUUCCTT

Supplement Table 2

Protein IDs	Protein names	Gene names	Fasta headers	Number of proteins	Peptides	Razor + unique peptides	Unique peptides	Sequence coverage	Mol. weight	Intensity	Intensity NT5DC2
[illegible]	Pre-B-cell leukemia transcription factor 2	hCG_2039588;PBX2	O7KZE5_HUMAN	6	1	1	1	4.3	37.643	0	0
[illegible]			A0A077H155_HUM	1	1	1	1	7	14.464	0	0
[illegible]	Leucine-rich repeat-containing protein 41	LRRC41	A0A087X0S7_HUM	6	1	1	1	2.5	56.4	0	0
[illegible]	Kinesin-like protein;Kinesin-like protein KIF1B	KIF1Bbeta;KIF1B	B4DMF3_HUMAN	9	1	1	1	1.7	99.185	0	0
[illegible]	Interferon-induced protein with tetratricopeptide repeats 2	IFIT2	A0A087X279_HUM	6	1	1	1	4	23.159	0	0
[illegible]	Phosphoinositide phospholipase C;Inactive phospholipase C-like protein 1	PLCL1	H3BUD4_HUMAN	4	1	1	1	1.8	114.69	0	0
[illegible]	Ras-related protein Rab-5B;Ras-related protein Rab-5A;Ras-related protein Rab-5C	RAB5A;RAB5B;RAB5	F8WCY6_HUMAN	15	1	1	1	17.7	6.8159	0	0
[illegible]	Tau-tubulin kinase 2	TTBK2	A0A0B4J292_HUM	3	1	1	1	2.3	50.077	0	0
[illegible]	Mediator of DNA damage checkpoint protein 1	MDC1	B4DYH4_HUMAN	13	1	1	1	1.1	178.74	0	0
[illegible]			A0A1X9I4Z1_HUM	2	1	1	1	19.1	10.866	0	0
[illegible]			A0A2H4G4A2_HUM	2	1	1	1	19.1	10.806	0	0
[illegible]	Lambda-crystallin homolog	HEL30;CRYL1	A0A2R8Y4K2_HUM	3	1	1	1	4.9	29.47	0	0
[illegible]	Unconventional myosin-IXa	MYO9A	H3BV44_HUMAN	4	1	1	1	0.9	157.88	0	0
[illegible]	Protein phosphatase 1B	PPM1B	B8ZZF0_HUMAN	7	1	1	1	4	33.232	0	0
[illegible]			A0A482E9C0_HUM	1	1	1	1	0.9	98.371	0	0
[illegible]			A0A5C2GCB0_HUM	1	1	1	1	15	11.745	0	0
[illegible]	Polypyrimidine tract-binding protein 2	PTBP2	B4DSI2_HUMAN	8	1	1	1	5.4	22.677	0	0
[illegible]	Protein very KIND	FLJ00378;KNDC1	Q8NF24_HUMAN	6	1	1	1	0.8	90.184	0	0
[illegible]	Ribosomal protein L19;60S ribosomal protein L19	RPL19	Q8WR8_HUMAN	6	1	1	1	5.2	20.819	0	0
[illegible]			A0A7S5EWB4_HUM	5	1	1	1	11.9	13.901	0	0
[illegible]			A0A7S5C028_HUM	1	1	1	1	16.3	13.831	0	0
[illegible]	Fibroblast growth factor;Fibroblast growth factor 5	FGF5	H0Y9E2_HUMAN	3	1	1	1	10.9	12.4	0	0
[illegible]	Dynein heavy chain 14, axonemal	DNAH14	A0A804HLD3_HUM	4	2	2	2	0.5	529.88	0	0
[illegible]	Kinesin-like protein;Kinesin-like protein KIF22	OBP-1;OBP-2;A-32	Q14984_HUMAN	8	1	1	1	10	12.121	0	0
[illegible]	S-adenosylmethionine mitochondrial carrier protein	SLC25A26	A0A8I5KTP7_HUM	4	1	1	1	10.4	14.328	0	0
[illegible]	Dynamin-3	DNM3	E5RIK2_HUMAN	6	1	1	1	2.9	50.732	0	0
[illegible]	Nebulin	NEB	A0A8I5KYD3_HUM	6	1	1	1	2.6	66.627	0	0
[illegible]	Golgin subfamily A member 4	GOLGA4	C9JHJ5_HUMAN	9	1	1	1	2.9	68.198	0	0
[illegible]	Thymosin beta-4;Hematopoietic system regulatory peptide	TMSB4X	A2VCK8_HUMAN	7	1	1	1	29.5	5.0526	0	0
[illegible]	E3 ubiquitin-protein ligase HECW1	NEDL1;HECW1	A4D1V5_HUMAN	2	1	1	1	0.7	177.16	0	0
[illegible]	Nucleolar protein 8	NOL8;DKFZp686P1	B4DM91_HUMAN	6	1	1	1	0.8	104.21	0	0
[illegible]	Zinc finger protein 468;Zinc finger protein 28	ZNF28;ZNF468	A8K1C6_HUMAN	5	1	1	1	4.3	54.401	0	0
[illegible]		KLRA1;LY49L	B4DJJ2_HUMAN	5	1	1	1	7.1	18.186	0	0
[illegible]	26S protease regulatory subunit 6B	PSMC4	A8K2M0_HUMAN	2	1	1	1	2.9	47.366	0	0
[illegible]	BTB/POZ domain-containing protein KCTD16	KCTD16	A8K8W2_HUMAN	2	1	1	1	3.7	49.084	0	0
[illegible]	Parvalbumin alpha	PVALB	B1AH72_HUMAN	4	1	1	1	10.3	8.4826	0	0
[illegible]	Extracellular sulfatase Sulf-2	SULF2	B1AMP9_HUMAN	2	1	1	1	6.9	21.499	0	0
[illegible]	Protein AKNAD1	AKNAD1	F8W8V4_HUMAN	6	1	1	1	6.2	16.614	0	0
[illegible]	Uncharacterized protein C9orf43	C9orf43	B4DX97_HUMAN	3	1	1	1	1.9	54.298	0	0
[illegible]			B3KT37_HUMAN	1	1	1	1	16.1	13.196	0	0
[illegible]	Chloride channel protein;H(+)/Cl(-) exchange transporter 3	CLCN3	B3KKK0_HUMAN	3	1	1	1	1.2	80.528	0	0
[illegible]	Vacuole membrane protein 1	VMP1;TMEM49	K7EPE7_HUMAN	5	1	1	1	4.4	25.147	0	0
[illegible]	Transcription termination factor 4, mitochondrial;mTERF domain-containing protein 2 processed	MTERF4	C9JX74_HUMAN	6	1	1	1	5.3	16.868	0	0
[illegible]	Bifunctional glutamate/proline--tRNA ligase;Glutamate--tRNA ligase;Proline--tRNA ligase	EPRS	H6WCP5_HUMAN	5	2	2	2	2.1	97.569	0	0
[illegible]	Smith-Magenis syndrome chromosomal region candidate gene 8 protein	SMCR8	B4DL51_HUMAN	2	1	1	1	1.2	75.31	0	0
[illegible]			B4DVW7_HUMAN	1	1	1	1	1.9	42.37	0	0
[illegible]	N-terminal kinase-like protein	SCYL1	B4DXZ7_HUMAN	5	1	1	1	5.1	39.647	0	0
[illegible]	Inversin	INVS	Q2M1I4_HUMAN	3	1	1	1	1	80.009	0	0
[illegible]			Q9P1D1_HUMAN	2	1	1	1	7	11.632	0	0
[illegible]			B7Z556_HUMAN	1	1	1	1	2.9	27.354	0	0
[illegible]		TBC1D2B	B9A6J8_HUMAN	1	1	1	1	0.8	109.95	0	0
[illegible]	Coiled-coil domain-containing protein 136	CCDC136	C9JU31_HUMAN	4	1	1	1	2.5	50.627	0	0
[illegible]		ST7	E7ENZ9_HUMAN	2	1	1	1	1.4	63.945	0	0
[illegible]	Toll-like receptor;Toll-like receptor 1	TLR1	Q6FI64_HUMAN	10	1	1	1	1.7	90.162	0	0
[illegible]	Serine protease inhibitor Kazal-type 5;Hemofiltrate peptide HF6478;Hemofiltrate peptide HF7665	SPINK5	E5RFU9_HUMAN	2	1	1	1	5.5	22.229	0	0
[illegible]	Transient receptor potential cation channel subfamily M member 2	TRPM2	Q5KTC1_HUMAN	4	1	1	1	1.6	147.24	0	0
[illegible]	Serine/threonine-protein kinase WNK1	WNK1	F5GWT4_HUMAN	2	1	1	1	0.4	225.5	0	0
[illegible]		RHD	G4XID0_HUMAN	1	1	1	1	62.5	2.9914	0	0
[illegible]	Centrosomal protein of 128 kDa	CEP128;LEDP/132	H0Y3E3_HUMAN	3	1	1	1	1.3	80.198	0	0
[illegible]	Tetratricopeptide repeat protein 22	TTC22	H0Y486_HUMAN	2	1	1	1	5.3	19.259	0	0
[illegible]	Adenosylhomocysteinase	AHCYL2	H0Y8B3_HUMAN	2	1	1	1	2.5	58.004	0	0
[illegible]		KRT1	H6VRG2_HUMAN	1	31	1	0	44.3	66.065	0	0
[illegible]	Protein O-GlcNAcase	MGEA5	H7C3X0_HUMAN	2	1	1	1	6.6	29.725	0	0
[illegible]		CPSF4L	H9KVA5_HUMAN	1	1	1	1	5.3	17.147	0	0
[illegible]		NAT9	J3QQJ2_HUMAN	1	1	1	1	9.8	9.2573	0	0
[illegible]	Small nuclear ribonucleoprotein Sm D2	SNRPD2	K7EJ85_HUMAN	2	1	1	1	14.8	6.1443	0	0
[illegible]		ACRC	L8E9I9_HUMAN	1	1	1	1	3.2	36.087	0	0
[illegible]			O14913_HUMAN	1	1	1	1	2.9	52.079	0	0
[illegible]	Putative 60S ribosomal protein L39-like 5;60S ribosomal protein L39	RPL39P5;RPL39	R39L5_HUMAN	2	1	1	1	19.6	6.3225	0	0
[illegible]	A-kinase anchor protein 17A	AKAP17A	X6RAJ1_HUMAN	2	1	1	1	2.2	51.506	0	0
[illegible]	Calmodulin-regulated spectrin-associated protein 2	CAMSAP2	CAMP2_HUMAN	1	1	1	1	0.8	168.09	0	0
[illegible]	Smoothelin-like protein 2	SMTNL2	SMTL2_HUMAN	1	1	1	1	3.3	50.196	0	0
[illegible]	Maestro heat-like repeat-containing protein family member 2B	HEATR7B2;MROH2	Q4G0Z6_HUMAN	2	1	1	1	3.1	37.576	0	0
[illegible]	Fibrous sheath-interacting protein 2	FSIP2	FSIP2_HUMAN	1	2	2	2	0.3	780.6	0	0
[illegible]	DENN domain-containing protein 2C	DENND2C	DEN2C_HUMAN	1	1	1	1	2.2	106.86	0	0
[illegible]		DKFZp781A0295	Q6AI58_HUMAN	1	1	1	1	1.6	120.31	0	0
[illegible]			Q6RGG0_HUMAN	1	1	1	1	6.4	14.632	0	0
[illegible]		DAZ1	Q6S4N0_HUMAN	1	1	1	1	52.4	2.4279	0	0
[illegible]	Probable helicase senataxin	SETX	SETX_HUMAN	1	1	1	1	0.5	302.88	0	0
[illegible]	Diencephalon/mesencephalon homeobox protein 1	DMBX1	DMBX1_HUMAN	1	1	1	1	2.9	41.198	0	0
[illegible]	Solute carrier family 25 member 40	SLC25A40	S2540_HUMAN	1	1	1	1	5.6	38.124	0	0
[illegible]	Ubiquitin carboxyl-terminal hydrolase 25	USP25	[illegible]	3	1	1	1	1.6	52.152	0	0
[illegible]	Integrator complex subunit 12	INTS12	INT12_HUMAN	1	1	1	1	1.5	48.807	0	0
[illegible]		gs78	Q96S04_HUMAN	1	1	1	1	2.4	35.799	0	0
[illegible]	Potassium voltage-gated channel subfamily F member 1	KCNF1	KCNF1_HUMAN	1	1	1	1	1.6	55.584	0	0
[illegible]	CGG triplet repeat-binding protein 1	CGGBP1	CGBP1_HUMAN	1	1	1	1	9	18.82	0	0
[illegible]	Vacuolar protein sorting-associated protein 51 homolog	VPS51	VPS51_HUMAN	1	1	1	1	1.7	86.041	0	0
[illegible]	Thyrotropin-releasing hormone-degrading ectoenzyme	TRHDE	TRHDE_HUMAN	1	1	1	1	0.9	117	0	0
[illegible]	Zinc finger protein 608	ZNF608	ZN608_HUMAN	1	1	1	1	0.8	162.21	0	0
[illegible]	Uncharacterized protein C5orf34	C5orf34	H0YAD0_HUMAN	2	1	1	1	7.9	14.628	770330	770330
[illegible]	Mitochondrial cardiolipin hydrolase	PLD6	PLD6_HUMAN	1	1	1	1	5.2	28.272	1877600	1877600
[illegible]		BSCL2	E9PPN5_HUMAN	17	1	1	1	39.1	2.5818	1954700	1954700
[illegible]	Protein arginine N-methyltransferase 1	PRMT1;HRMT1L2	A0A3S6H7Y8_HUM	43	1	1	1	12.1	12.303	2145600	2145600
[illegible]	Cancer-related nucleoside-triphosphatase	NTPCR;C1orf57	Q5TDF0_HUMAN	3	1	1	1	4.8	25.072	2712500	2712500
[illegible]	Phosphatidylglycerophosphatase and protein-tyrosine phosphatase 1	PTPMT1	B4DGK8_HUMAN	3	1	1	1	8.8	15.761	3280400	3280400
[illegible]	Peptidyl-prolyl cis-trans isomerase FKBP4;Peptidyl-prolyl cis-trans isomerase FKBP4, N-terminally proc	FKBP4	B2R9U2_HUMAN	8	1	1	1	3.1	51.834	3327200	3327200
[illegible]	Probable ATP-dependent RNA helicase DDX5;Probable ATP-dependent RNA helicase DDX17	DDX5;DKFZp686J01	A0A0U1RQJ0_HUM	17	1	1	1	21.4	5.9768	3563000	3563000
[illegible]	Proteasome subunit beta type-5	PSMB5	PSB5_HUMAN	3	2	2	2	8.7	28.48	3626800	3626800
[illegible]	Nucleolar MIF4G domain-containing protein 1	NOM1	NOM1_HUMAN	1	1	1	1	1.3	96.256	3812200	3812200
[illegible]	Calcium-transporting ATPase;Calcium-transporting ATPase type 2C member 1	ATP2C1	H0Y9V7_HUMAN	7	2	2	2	2.3	98.938	3854400	3854400
[illegible]	Alpha-1,6-mannosyl-glycoprotein 2-beta-N-acetylglucosaminyltransferase	MGAT2	B4DDK9_HUMAN	2	1	1	1	3.5	52.297	4394900	4394900
[illegible]	60S ribosomal protein L18a	RPL18A	Q32XH3_HUMAN	8	1	1	1	9.6	15.791	4549600	4549600
[illegible]	Phospholipase ABHD3	ABHD3	J3KTE1_HUMAN	2	1	1	1	3.7	40.393	4599500	4599500
[illegible]	Prohibitin-2	PHB2	F5H3X6_HUMAN	3	1	1	1	4.9	[illegible]	5020200	5020200
[illegible]	Epoxide hydrolase 4	EPHX4	EPHX4_HUMAN	1	1	1	1	3	42.324	5076100	5076100
[illegible]	GPN-loop GTPase 1	MBDin;GPN1	Q53RZ9_HUMAN	2	1	1	1	2.9	41.74	5083100	5083100
[illegible]	L-lactate dehydrogenase;L-lactate dehydrogenase A chain;L-lactate dehydrogenase C chain;L-lactate	LDHC;LDHB;HEL-S-	F5H5G7_HUMAN	19	1	1	1	6.6	20.008	5252600	5252600
[illegible]	Long-chain-fatty-acid--CoA ligase 1	ACSL1	H0Y9U7_HUMAN	8	1	1	1	3.7	32.809	5582400	5582400
[illegible]	Enolase;Alpha-enolase	ENO1	A4QMW8_HUMAN	5	1	1	1	11.1	14.536	5600600	5600600
[illegible]	[illegible]	[illegible]	[illegible]	[illegible]	[illegible]	[illegible]	[illegible]	[illegible]	[illegible]	[illegible]	[illegible]
