# Supplementary material for: NT5DC2 inhibits ferroptosis by stabilizing ACSL3 in bladder cancer
Source: Cell Death Discov. 2026 Apr 14;12:235. doi: 10.1038/s41420-026-03091-1 (PMC13184144; doi:10.1038/s41420-026-03091-1)

Figure 2B:

NT5DC2

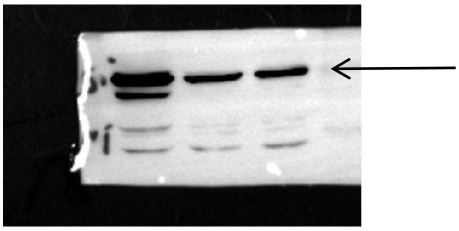

Actin

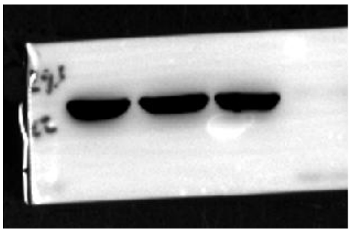

Figure 2H:

NT5DC2

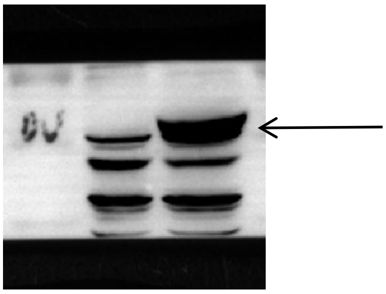

Actin

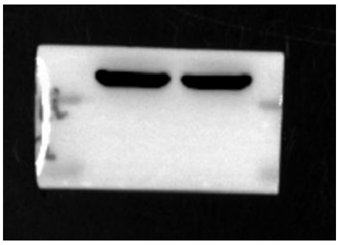

Figure 3B

NRF2

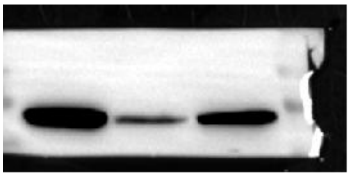

GPX4

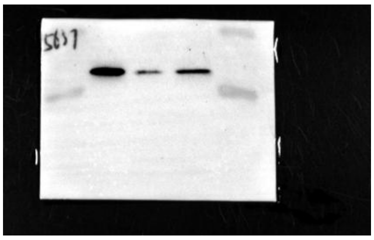

Ferritin

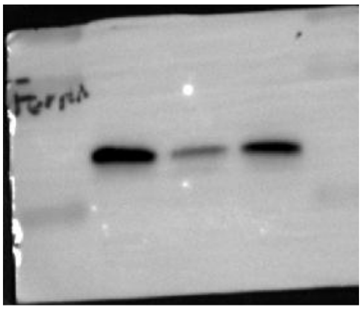

NT5DC2

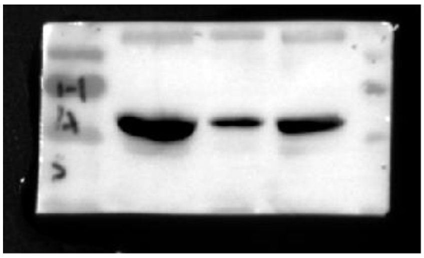

Actin

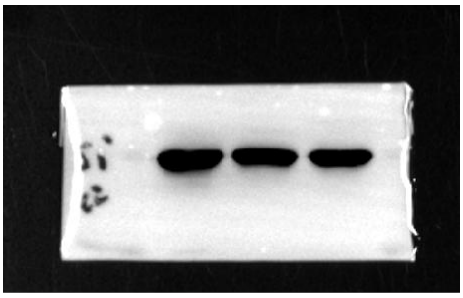

Figure3G

NRF2

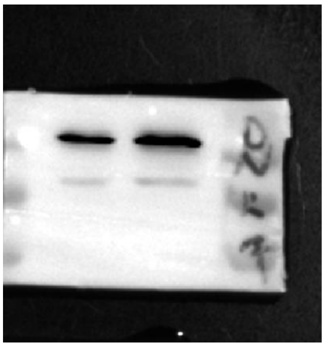

GPX4

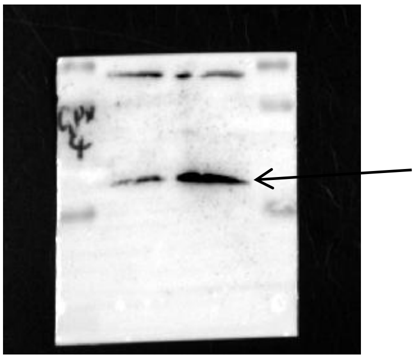

Ferritin

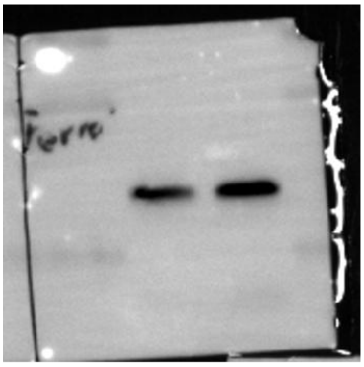

NT5DC2

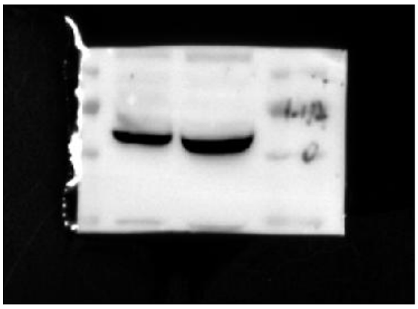

Actin

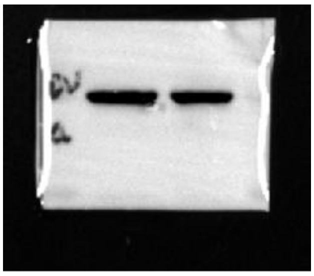

Figure5B

IP-Flag

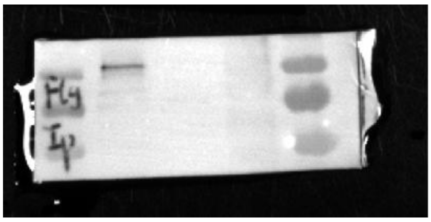

IP-HA

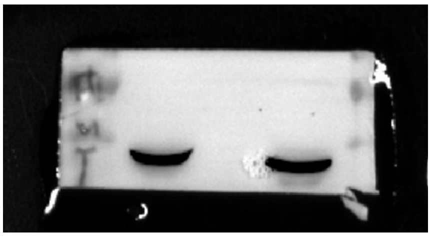

Input-Flag

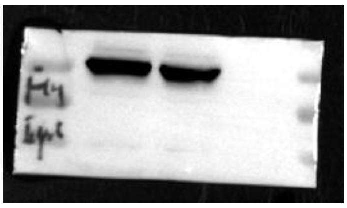

Input-HA

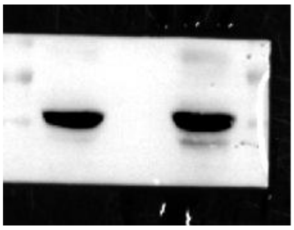

Figure 5C

IP-HA

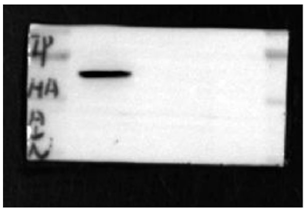

IP-Flag

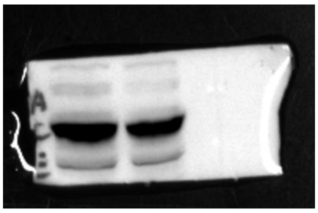

Input-HA

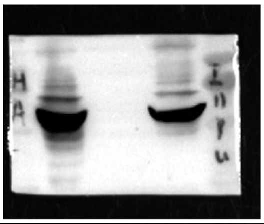

Input-Flag

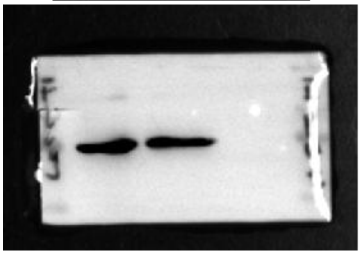

Figure 5D

5637-NT5DC2

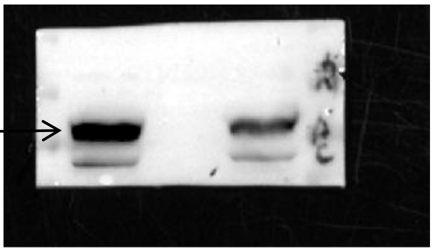

5637-ACSL3

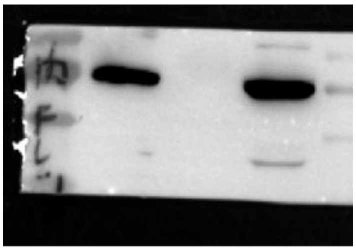

UMUC3-NT5DC2

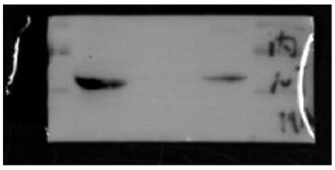

UMUC3-ACSL3

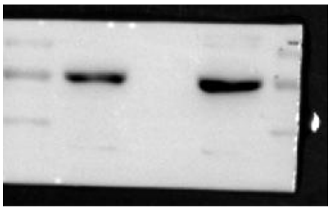

Figure 5G

IP-Flag

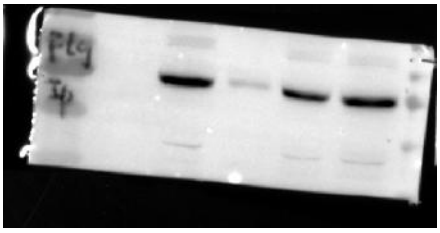

IP-HA

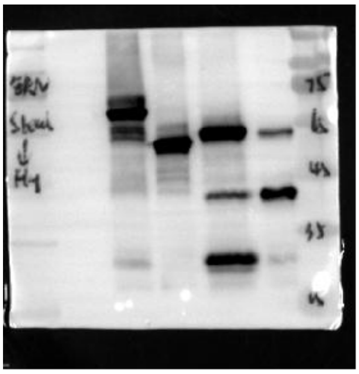

Input-HA

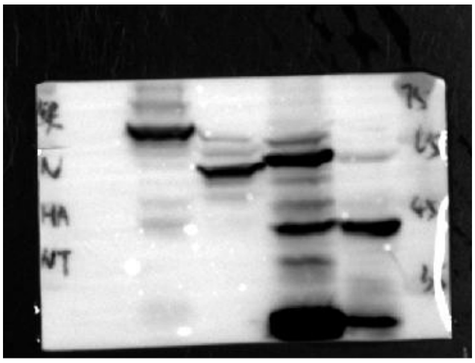

Input-Flag

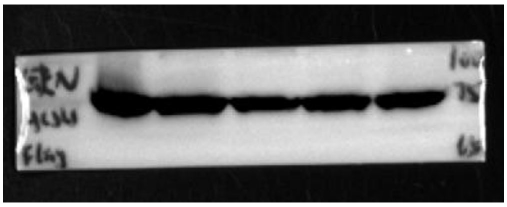

Figure 5H

IP-HA

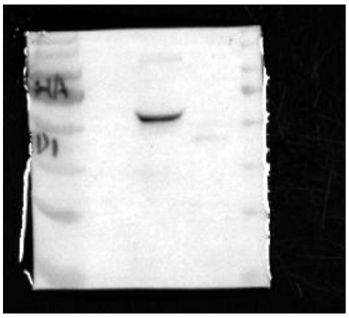

Ip-Flag

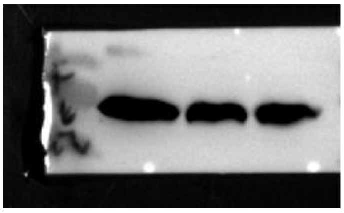

Input-HA

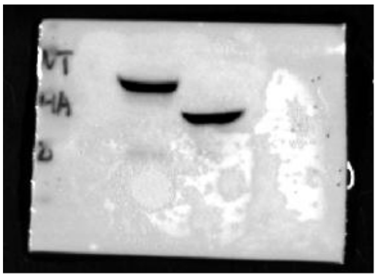

Input-Flag

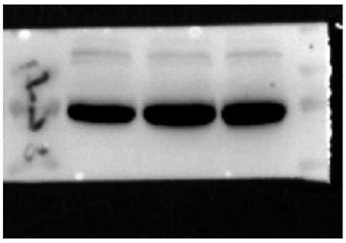

Figure 5I

IP-HA

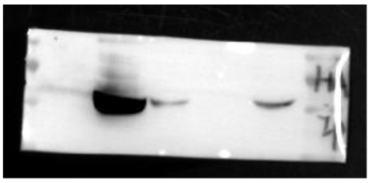

Ip-Flag

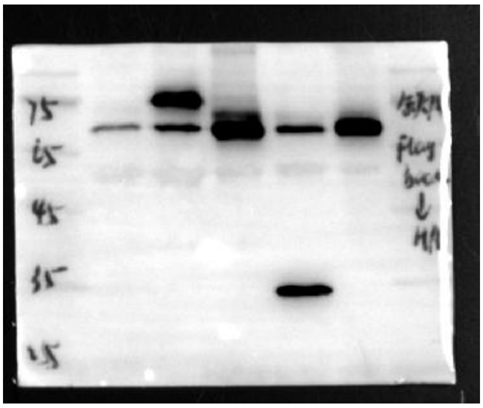

Input-Flag

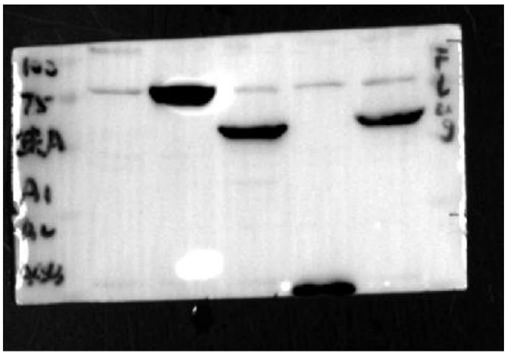

Input-HA

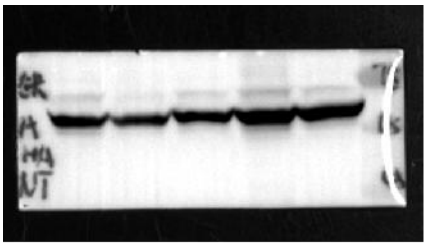

Figure 5J

IP-Flag

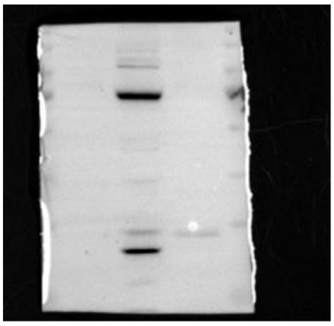

IP-HA

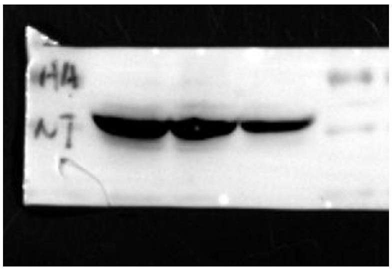

Input-Flag

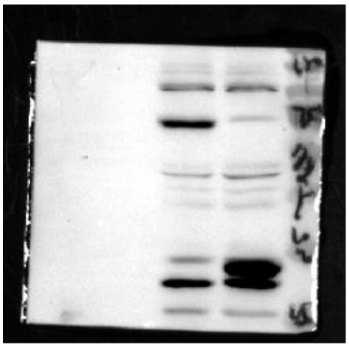

Input-HA

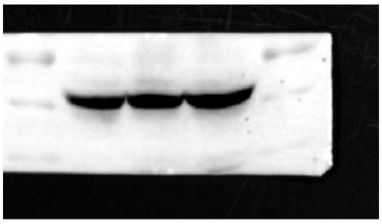

Figure 6A

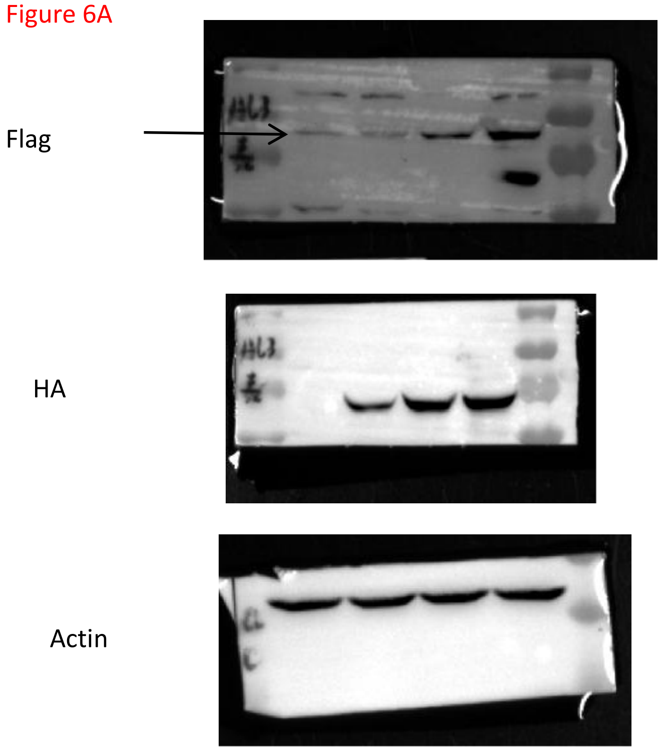

Figure 6B

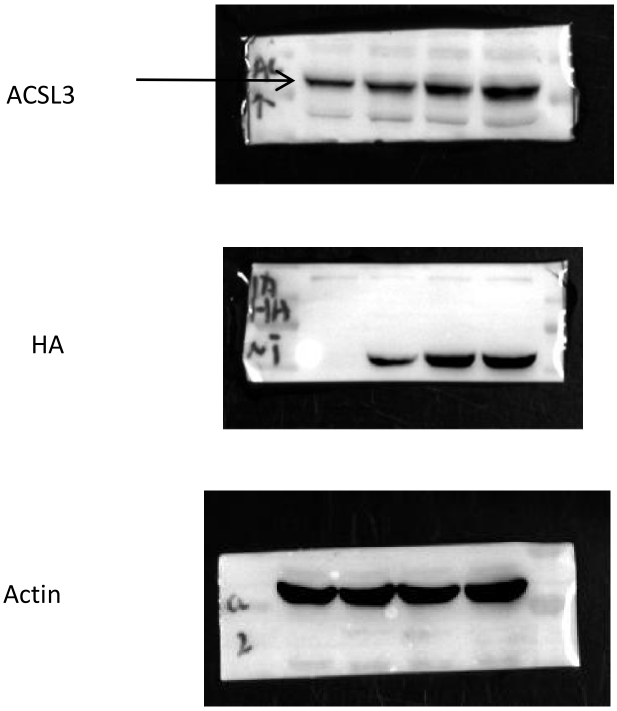

Figure 6C

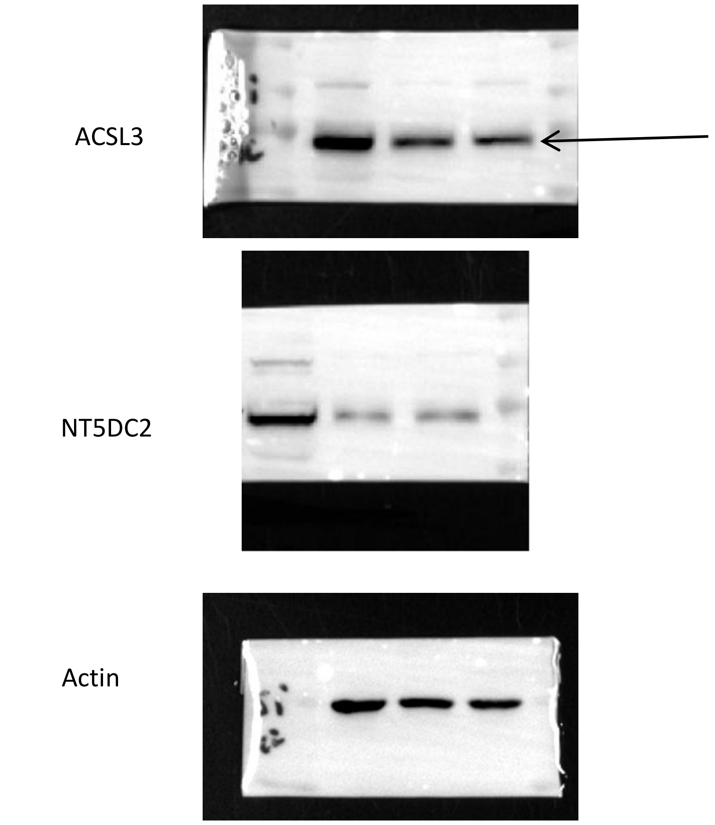

Figure 6D

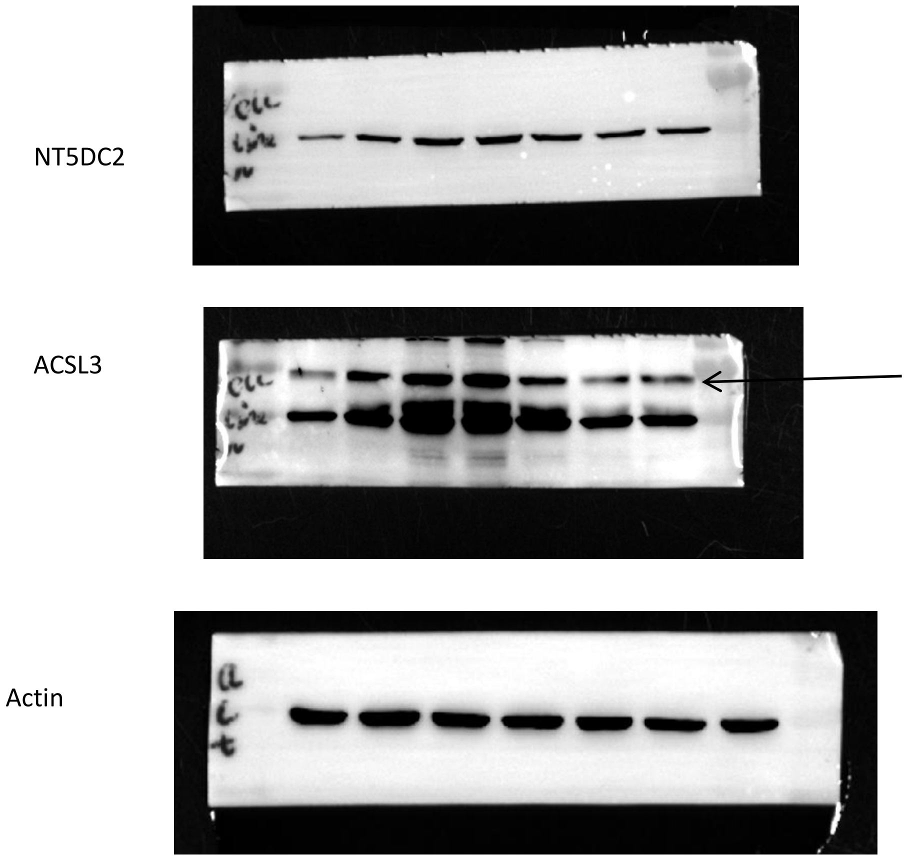

Figure 6G

Flag

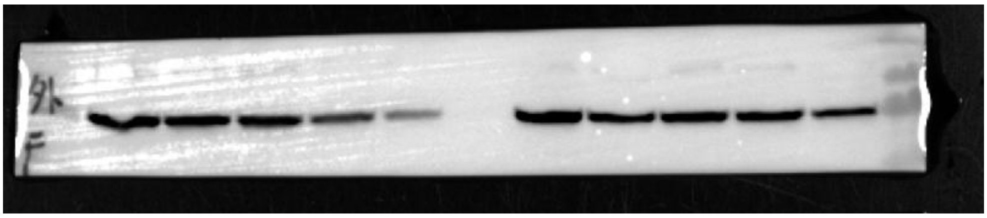

HA

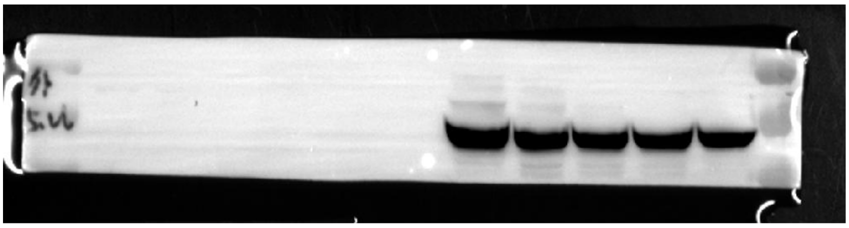

Actin

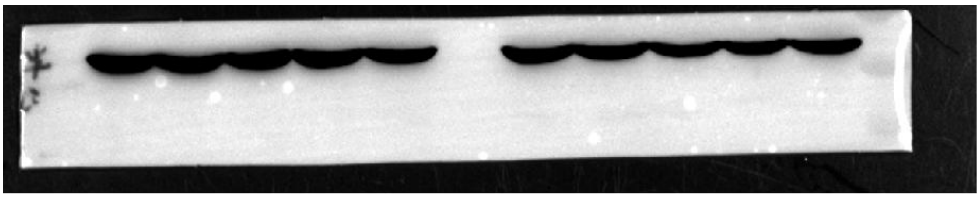

Figure 6H

ACSL3

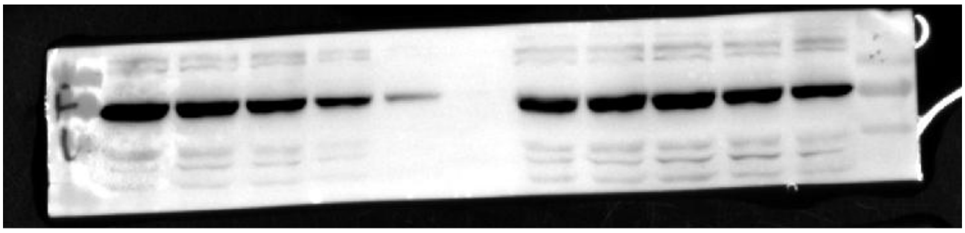

HA

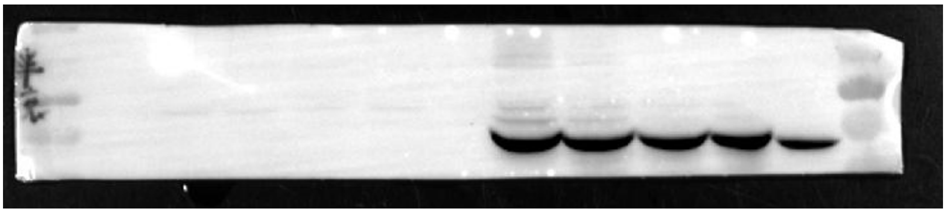

Actin

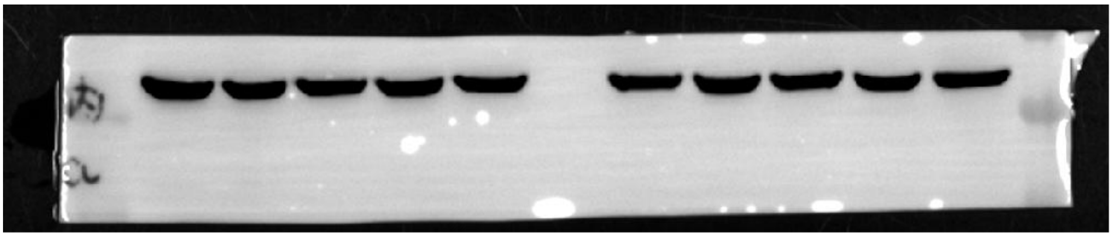

Figure 6I

ACSL3

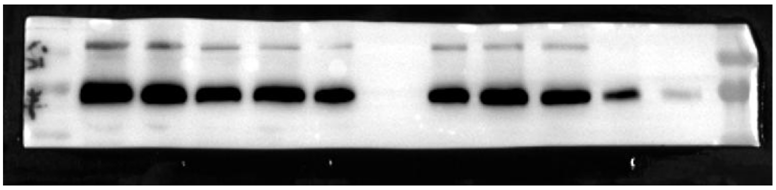

NT5DC2

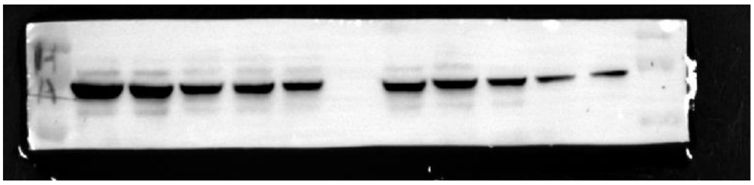

Actin

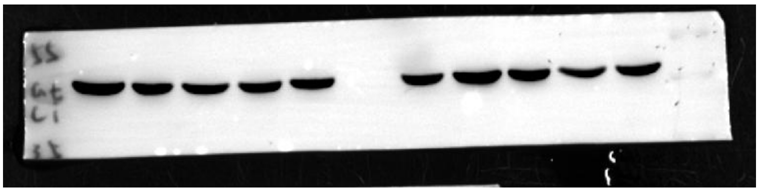

Figure 6J

NT5DC2

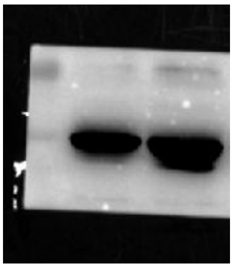

ACSL3

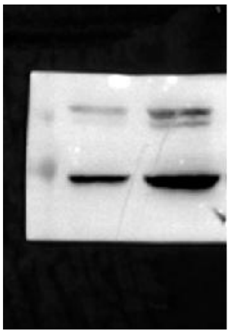

Actin

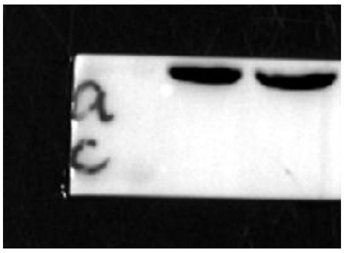

Figure 6K

IP-MYC

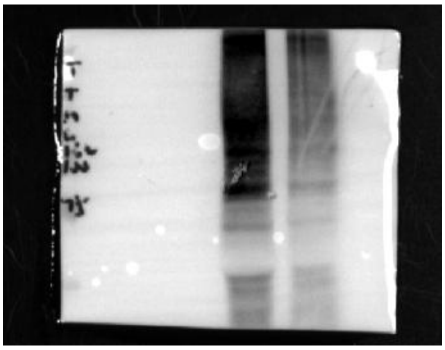

Ip-Flag

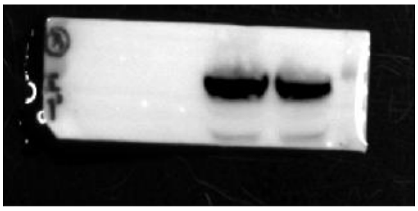

Input-HA

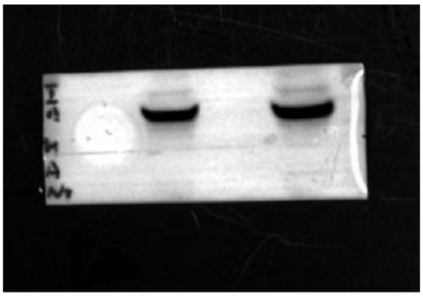

Input-Flag

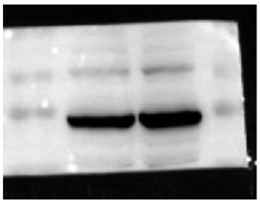

Input-MYC

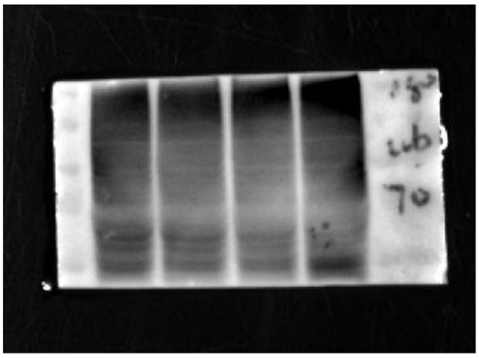

Figure 6L

IP-MYC

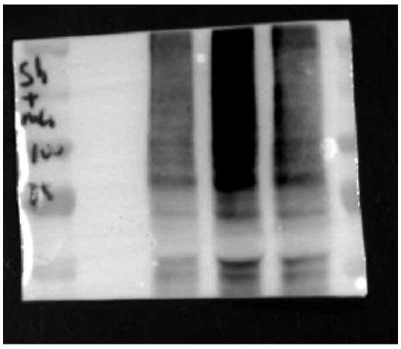

IP-Flag

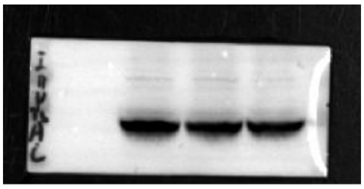

NT5DC2

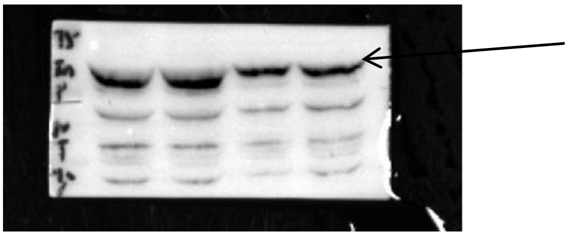

Input-Flag

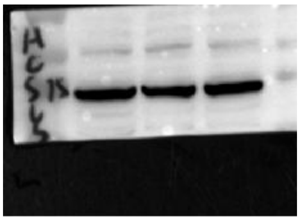

Input-MYC

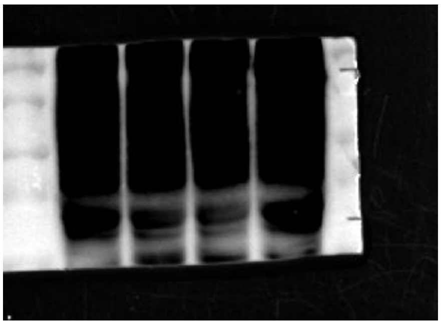

Figure 7A

ACSL3

Actin

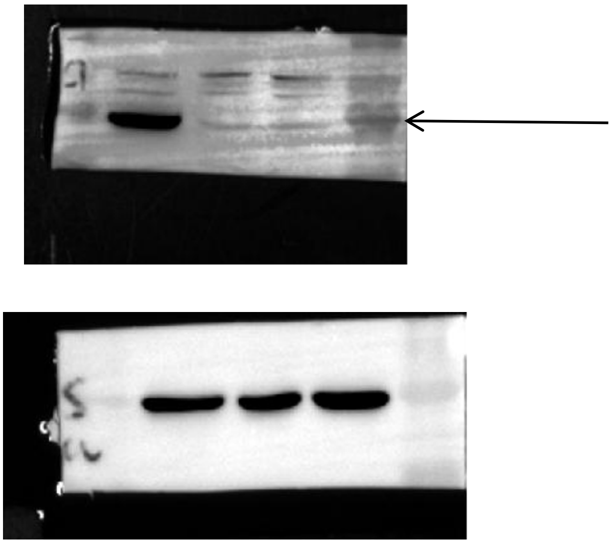

Figure 7F

ACSL3

Actin

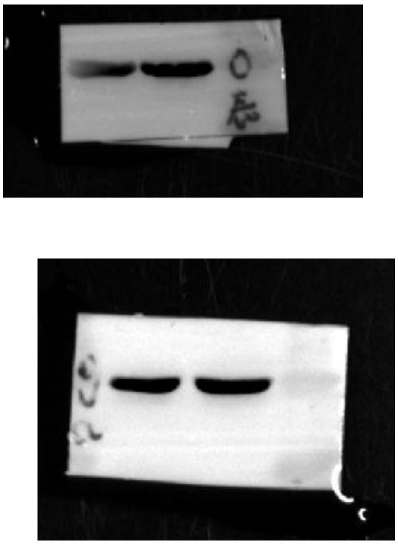

Figure 8A

NT5DC2

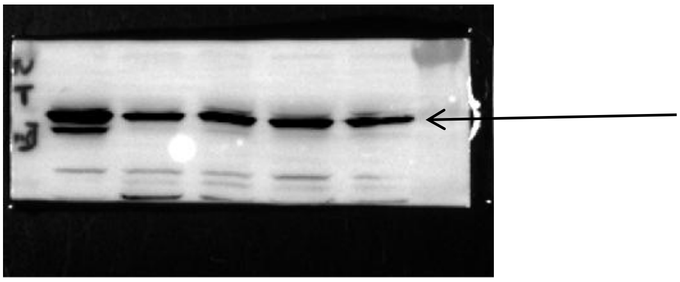

ACSL3

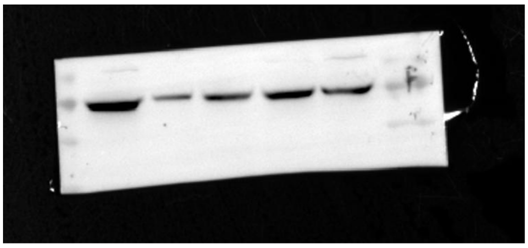

Actin

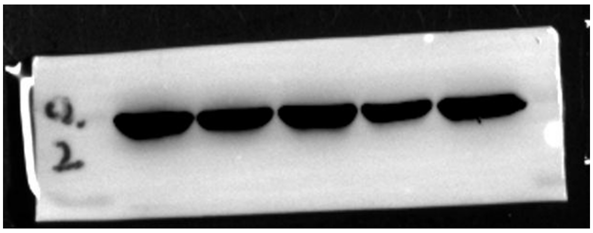

Figure 8F

NRF2

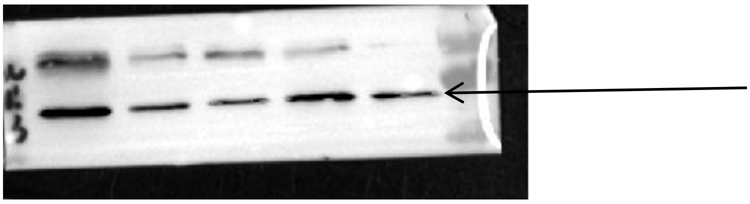

GPX4

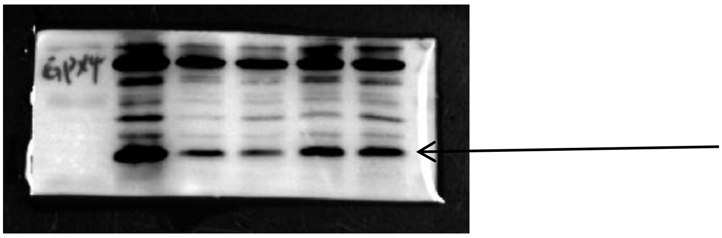

Actin

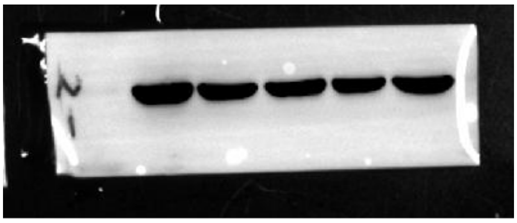

Figure 9B

NT5DC2

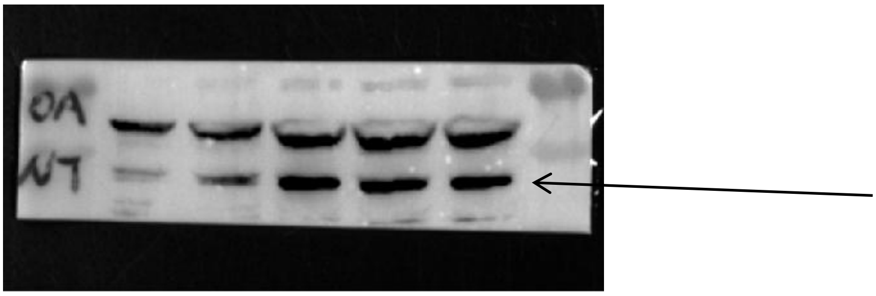

ACSL3

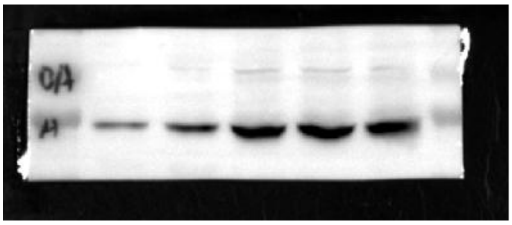

Actin

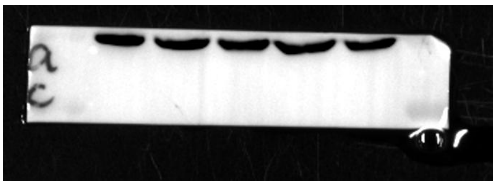

Figure 9C

NT5DC2

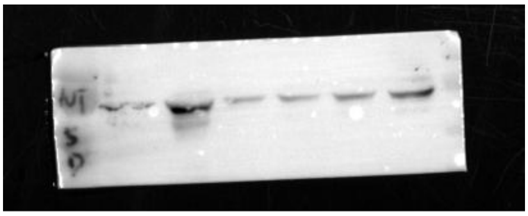

ACSL3

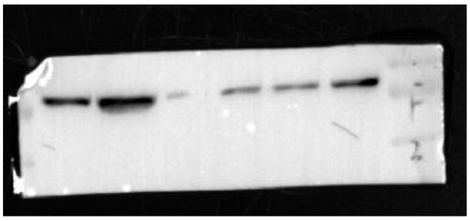

Actin

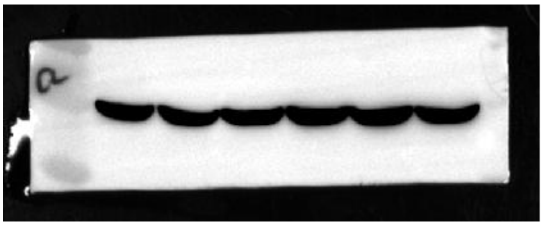

Figure Supplymentary 4A

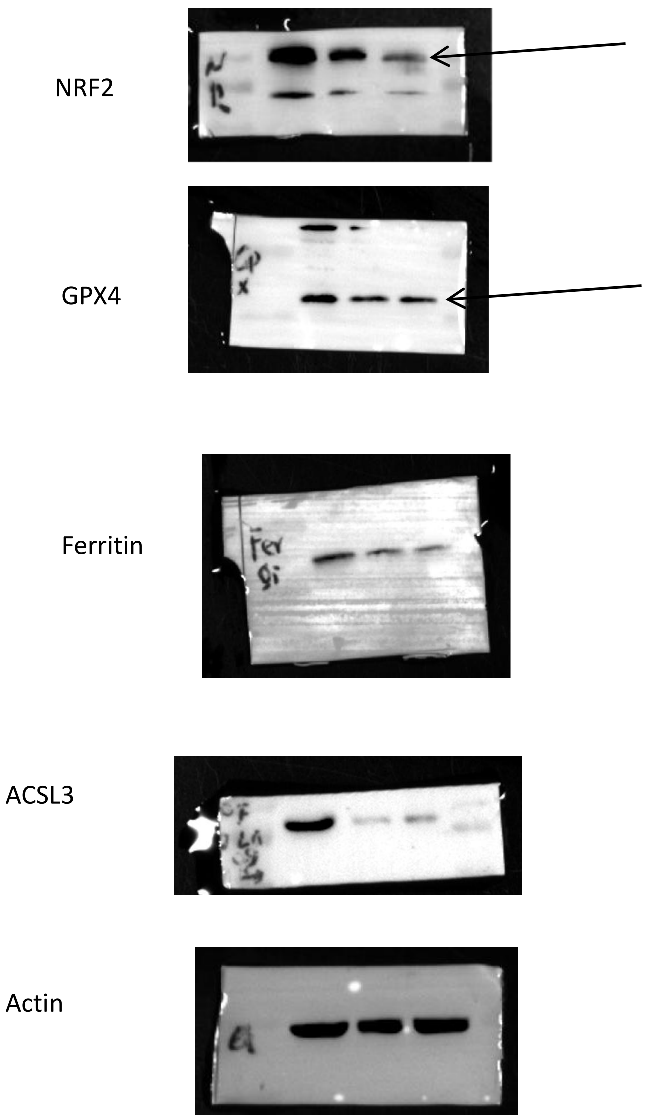

Figure Supplymentary 4F

NRF2

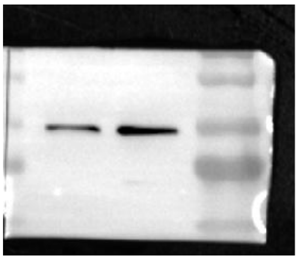

GPX4

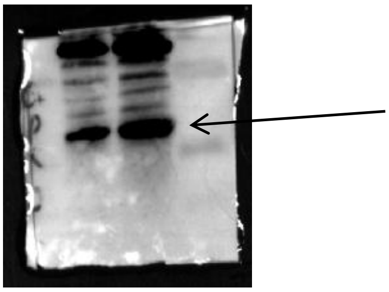

Ferritin

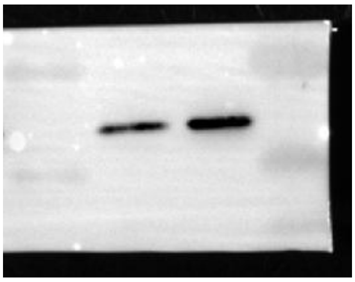

ACSL3

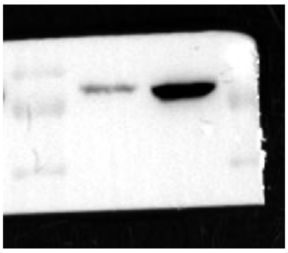

Actin

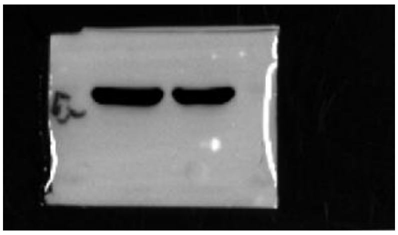

Supplement: Supplementary file 2 — WB-Data [file 41420_2026_3091_MOESM2_ESM.pdf]
